# Catalyst free PET and PEF polyesters using a new traceless oxalate chain extender†

**DOI:** 10.1039/d4gc02791d

**Published:** 2024-08-16

**Authors:** Kevin van der Maas, Daniel H. Weinland, Robert-Jan van Putten, Bing Wang, Gert-Jan M. Gruter

**Affiliations:** a Van't Hoff Institute of Molecular Sciences, University of Amsterdam Science Park 904 1098 XH Amsterdam The Netherlands G.J.M.Gruter@uva.nl; b Avantium Chemicals BV Zekeringstraat 29 1014BV Amsterdam The Netherlands

## Abstract

Plastic material performance is strongly correlated to the polymer's molecular weight. Obtaining a sufficiently high molecular weight is therefore a key goal of polymerization processes. The most important polyester polyethylene terephthalate (PET) and the new polyethylene furanoate (PEF) require metal catalysts and time-consuming production processes to reach sufficiently high molecular weights. Metal catalysts, which are typically antimony or tin for polyesters, end up in the plastic products which may result in sustainability and ecological challenges. When the less reactive comonomer isosorbide is introduced to produce (partly) biobased materials with enhanced thermal properties, such as polyethylene-*co*-isosorbide furanoate (PEIF), reaching high enough molecular weight becomes even more challenging. This study presents an easily implementable approach to produce high molecular weight PET and PEF polyesters and their isosorbide copolyesters PEIT and PEIF by coupling lower molecular weight polymer chains by the reactive diguaiacyl oxalate (DGO) chain extender. DGO is so reactive, that the use of metal catalysts can be completely avoided and it helps avoiding an extra solid-state polymerization step. In addition, DGO distinguishes itself from typical chain extenders by its ability to be completely removed from the resulting polymer, thereby avoiding the inherent drawbacks associated with typical chain extenders.

## Abstract 2

When producing PET the metal catalyst (typically Sb) remains in the polymer. This raises concerns as our food and the air we breathe is contaminated with PET microplastics. Here we report the production of metal-free PET using a small amount of DGO.

## Graphical abstract

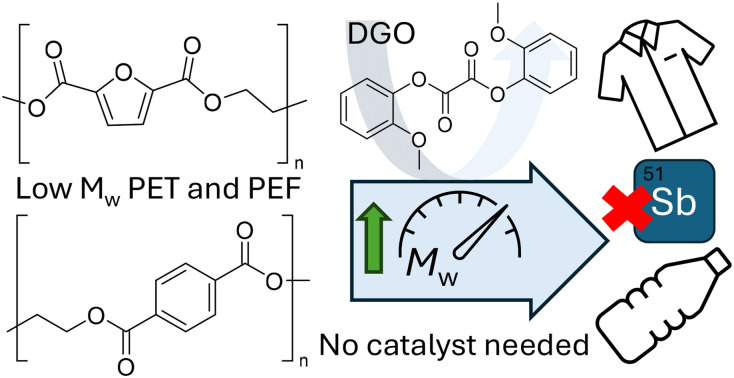

## Introduction

With more than 70 million tons per year of polyethylene terephthalate (PET) manufactured worldwide, PET is the most important commercial polyester today.^1–3^ PET finds application in numerous products, especially packaging and textile products such as bottles, sheets, carpets and clothes.^4^ In Europe, PET accounts for roughly 16% of the plastic consumption.^5^ Polyethylene furanoate (PEF) is considered as one of the most promising renewable future polyester and PEF is expected to become commercially available in 2024.^6–8^ Compared to PET, PEF shows improved barrier-, thermal- and mechanical performance, allowing for thinner packaging with improved performance.^6^

Melt polycondensation is the most common synthetic method for large-scale production of polyesters.^9^ It is essentially a reversible esterification reaction with a relatively low equilibrium constant and relies on the removal of the condensation products (typically water) to reach sufficient molecular weight. Material performance of plastics is strongly correlated to the molecular weight of the polymer: insufficient molecular weight could lead to appliance failure.^10^ As the molecular weight increases, the viscosity of the melt also increases, making it more difficult to remove the condensation products. Eventually, this becomes a limiting factor for the molecular weight.^11^ To further enhance the removal of condensation products, higher temperature, longer reaction time and improved reactor designs are used.^12^ However, these harsher conditions also contribute to unwanted side reactions, which change the physical properties of the material and will result in product coloration. Decarboxylation of PEF's monomer furan-2,5-dicarboxylic acid (FDCA) and acetaldehyde formation from the ethylene glycol moiety are examples of undesired side reactions often seen with the production of PEF and PET under harsher conditions.^9,13^ Also metal catalysts are added to accelerate the polymerization process. The vast majority of global PET production (>90%) relies on antimony trioxide as the catalyst. However, concerns have been raised about the safety of antimony, given its classification as a heavy metal, particularly when it is utilized in products that come into contact with water, beverages, pharmaceuticals, and food items.^14–20^ Additionally, PET fibres (typically containing Sb catalyst) have been reported in a wide range of human foods and beverages, including seafood, drinking water, beer, salt and sugar.^17^

For PET, it is difficult to obtain a number average molecular weight (*M*_n_) higher than 20 kilodalton (kDa), equal to an intrinsic viscosity (IV) of ∼0.60 dL g^−1^, without additional processes after the melt polymerization.^21^ This molecular weight range is sufficient for applications demanding lower molecular weight, such as textiles. To obtain higher molecular weights necessary for bottle applications (0.73–0.85 dL g^−1^) and industrial yarns (>1.2 dL g^−1^) an additional solid-state polymerization (SSP) is typically required.^22,23^ In SSP, polymer pellets are heated above the glass transition temperature (*T*_g_), but below the melting point (*T*_m_), while being rotated under a nitrogen flow or reduced pressure. Due to the low mobility of the polymer end groups and condensate in the solid state, it is a time-consuming process and therefore energy consuming and expensive.^24^ Typically, for PET SSP takes more than 8 hours.^25^

When less reactive diols, like isosorbide, are introduced to produce *e.g.* polyethylene-*co*-isosorbide terephthalate (PEIT) and polyethylene-*co*-isosorbide furanoate (PEIF), it becomes even more challenging to obtain sufficient molecular weight.^26–29^ Isosorbide is a commercial biobased building block produced from glucose *via* sorbitol.^30^ Incorporation of isosorbide is desired due to its positive effect on thermomechanical stability and mechanical performance, which enables the production of high-performance biobased materials. DURABIO and ECOZEN are examples of commercially available isosorbide-based engineering polymers and can be found in smart phone screens, car dashboards, sports drink bottles and food containers.^30–32^ Isosorbide is less reactive due to its secondary alcohol groups and melt polycondensation becomes considerably more difficult with increasing isosorbide content. Additionally, the crystallinity is lost with isosorbide contents above 15 mol% (relative to total diol), which makes SSP impossible, as amorphous polymer pellets will stick and clump together at temperatures above their *T*_g_.^33^ These limitations hamper the growth of the isosorbide-based polyesters market.

An alternative route for producing high molecular weight polymers is by using a so-called chain extender after melt polycondensation.^22,25,34^ Chain extenders are very reactive molecules that function as a coupling agent by reacting with the remaining functional chain ends to increase the molecular weight. When combined with melt polycondensation, only a small amount of chain extender is required, as already a considerable chain length can be obtained by melt polycondensation. In this manner the final stage and most difficult part of the polymerization can be easily overcome by the high reactivity of the chain extender, which leads to considerably less time and less harsh conditions to obtain high molecular weights. Additionally it can make SSP redundant.^22^ The combination of these factors likely reduces the process costs significantly. Chain extenders are also attractive for recycling or up-cycling polymers, due to the low cost and easy use.^35,36^

Various chain extenders have been used for polyesters, for example: bis-oxazolines, pyromellitic dianhydride, organic phosphites, di-isocyanates, di-epoxides, carbonyl biscaprolactam, diphenyl carbonate, diphenyl esters, bisketenimines, bislactams.^10,22,35,37–44^ However, the use of chain extenders also comes with significant drawbacks; often, side reactions occur, such as crosslinking or chain scission, which changes the physical properties of the final polymer. After each chain extension, the end of life and recycling of the polymer becomes more complicated.^36^ Furthermore, the chain extender residues that are incorporated into the polymer backbone often influence the properties of the resulting polymer and some chain extenders are considerably toxic, on their own, or as residue in the final polymer, which excludes their use in food-grade applications.^10,45^ These drawbacks are probably the reason that chain extenders are rarely used today in standard commercial polyester production.

**Fig. 1:**
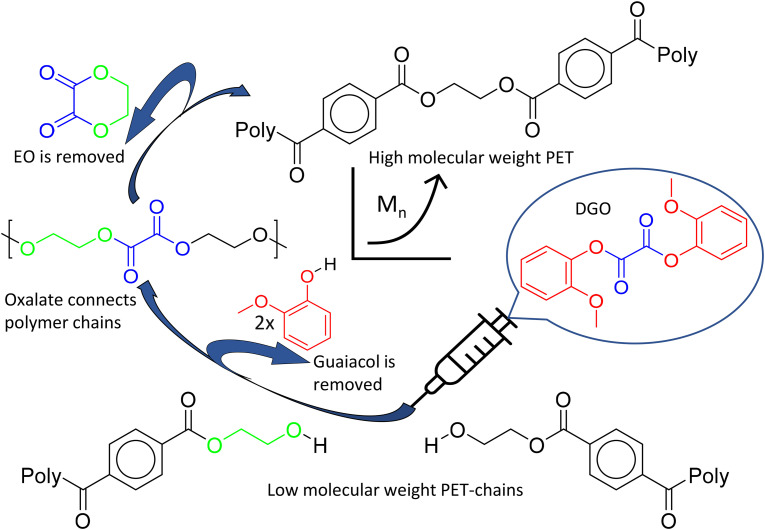
Schematic illustration of the molecular weight boosting of PET. Starting at the bottom, a low molecular weight polymer of PET is reacted with a small amount of DGO to produce a high molecular weight PET. DGO is a very reactive difunctional molecule, able to link residual alcohol end-groups together by transesterification. Above a certain temperature, the resulting oxalate in the polymer chain readily forms ethylene oxalate, a six membered ring molecule with its ethylene glycol neighbor. All DGO components, guaiacol and EO, are then removed from the reactor at the process temperature and reduced pressure. For a more detailed reaction mechanism of the ethylene oxalate formation, see the ESI overview S1.†

Our previous research on isosorbide based polyesters revealed that diaryl oxalates are very effective in polyesterification reactions due to their high reactivity, caused by the leaving group properties of aryl groups and the fact that the carboxyl groups of oxalate are vicinal.^46^ Especially the diguaiacyl ester (DGO) is highly reactive, due to the steric hindrance caused by the methoxy group in the *ortho* position, impeding the reverse reaction of guaiacol with the polymer chain. Another interesting characteristic of oxalate is its ability to create a six-membered ring when combined with ethylene glycol in the polymer, leading to the formation of ethylene oxalate (EO).^44,47,48^ This opens a route to use the high reactivity of oxalate and at the same time remove it from the polymer structure by ring formation, see Fig. 1. This shows potential for chain extender applications, free from the inherent drawbacks of commonly used chain extenders. Therefore this research is focused on the use of diguaiacyl oxalate as a traceless chain extender for catalyst free polyester.

## Results

### Presence and removal of oxalate in PET

**Fig. 2:**
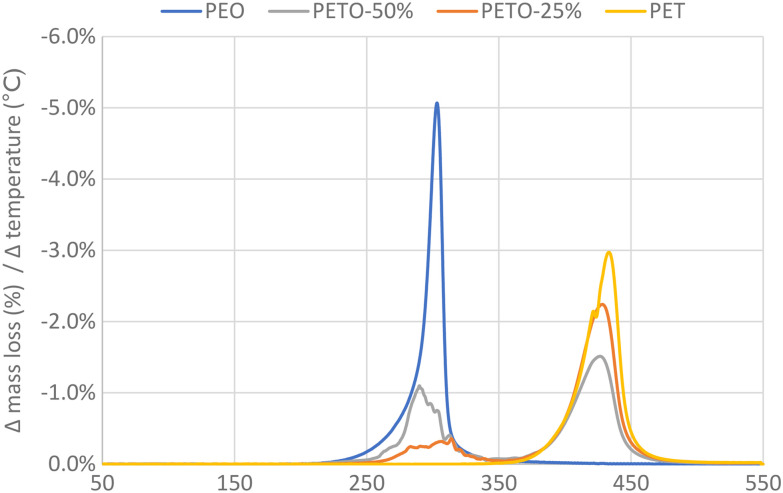
Oxalate removal upon heating (TGA). PET, polyethylene oxalate (PEO) and PET-oxalate copolymers (PETO) were subjected to heating at 10 °C min^−1^. The derivative of mass loss (%) with respect to temperature is given. The mentioned percentages of PETO represent the amount of oxalate in the polymer, expressed as fraction of the total acid (oxalate + terephthalate).

First oligo-ethylene terephthalate (*e.g.* bis(2-hydroxyethyl) terephthalate) was reacted with a stoichiometric amount of DGO to obtain the copolymer polyethylene terephthalate-*co*-oxalate (PETO), see Experimental section **[A]** for the synthesis of DGO and for the synthesis of PETO see **[B]** and **[C]**. When the conditions are kept sufficiently mild, the oxalate will remain in the polymer: with the help of ^1^H and ^13^C NMR analysis, the oxalate could be identified and quantified (Fig. S1 and S2†). Obtaining high molecular weights under these mild conditions, such as low polymerization temperature (<200 °C) and no catalyst, could be achieved using DGO. If harsher conditions were used (>200 °C), oxalate was eliminated from the polymer. This makes it quite challenging to produce PETO with significant oxalate content. From the synthesis of poly(ethylene oxalate) it is well known that ethylene glycol and oxalate can form the six membered ring compound ethylene oxalate (EO).^47–49^ When PETO was subjected to a temperature ramp (using TGA), it could be clearly proven that also in our case, the oxalate was eliminated from the polymer matrix as EO (Fig. 2). ^1^H and ^13^C NMR analysis of a sample of the sublimation product confirmed that ethylene oxalate was formed (Fig. S3†). These results indicated that DGO could be used as an effective chain extender and that its carbon content could be removed relatively easily from the polymer backbone.

### PET molecular weight boosting

**Fig. 3:**
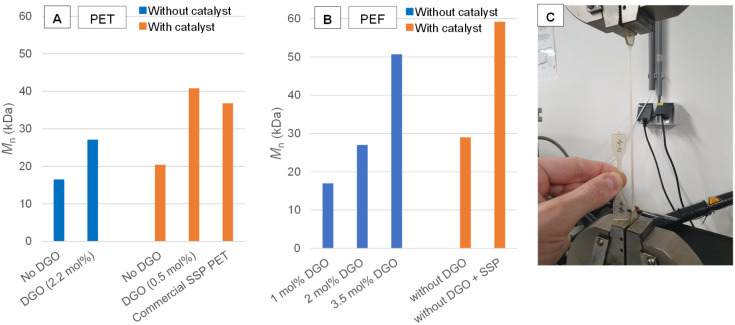
The effectiveness of the DGO booster on the molecular weight of PET and PEF. (A) *M*_n_ obtained from PET DGO-assisted melt polycondensations; the blue bar indicates that no catalyst was added and the orange bar indicates that a catalyst was added. The reported percentages are the amounts of oxalate added relative to the TPA content, see Fig. S4 for more detailed SEC results. (B) *M*_n_ obtained from PEF DGO-assisted melt polycondensations; the blue bar indicates that no catalyst was added and the orange bar indicates that a antimony catalyst was added. The reported percentages are the amounts of oxalate added relative to the FDCA content, see Fig. S4† for more detailed SEC results. (C) Catalyst free DGO-assisted PET tensile bar being tested, with a virgin tensile bar next to it.

This opened up a number of promising applications for this chemistry. DGO-assisted PET syntheses were performed under different conditions in order to evaluate the potential of this method. Fig. 3A provides an overview of the results. In the conducted experiments, catalyst-free polymerizations were carried out, which typically results in the formation of low molecular weight polymers,^50^ see Experimental section [D], [E] and [F] for the PET synthesis. However, when catalyst free PET was produced by the minor addition of 2.2 mol% DGO during the polycondensation, average molecular weight (*M*_n_) values as high as 27 kDa were obtained. These numbers are higher than those obtained from regular PET polycondensation with catalyst (20 kDa; ∼0.60 dL g^−1^), but not as high as those found after SSP (37 kDa; ∼0.8 dL g^−1^), yet they can be sufficient for certain applications.^51^ When the polycondensation with a titanium catalyst is combined with subsequent minor addition of DGO (0.5 mol%), the *M*_n_ can be boosted to very high values (41 kDa), normally only obtainable with SSP. To verify no additional metals were introduced by the addition of DGO, Inductively Coupled Plasma (ICP) analysis was performed and confirmed no common metals used in polymerization chemistry were detected above the limit of detection (see ESI Overview S2†). To further demonstrate that the catalyst free DGO-assisted PET is useful in an application setting, the resin was further processed into tensile bars (see Fig. 3C for a picture) and compared to non-boosted and commercial PET. The tensile tests show that the non DGO-assisted PET without catalyst has poor mechanical performance with brittle fractures (elongation at break 5.5% ± 2.0). In contrast, the DGO-assisted PET shows good mechanical performance similar to commercial PET (elongation at break 445.8% ± 169.5). See ESI Overview S3† for the tensile test results and other physical characterization.

### PEF molecular weight boosting

As this method showed promise in PET synthesis, it was logically also applied to PEF synthesis. In Fig. 3B the results are shown for the stepwise addition of DGO to a melt of PEF without metal catalyst, see Experimental section **[G]** and **[H]** for the PEF synthesis. DGO was incrementally added to the molten mixture containing the low molecular weight PEF, which was then left to undergo a 30-minute reaction before sampling. The NMR spectra of these samples showed that DGO effectively reacted with the ethylene glycol end-groups and reduced their amount after each addition (Fig. S5†). This was in line with the SEC results, as the molecular weight increased after each addition. *M*_n_ values as high as 51 kDa were obtained using 3.5 mol% relative to the FDCA content. This clearly exceeds the *M*_n_ of the regular polycondensation in the presence of Sb catalyst and provides values close to what normally can only be obtained after SSP.

One interesting observation from the DGO-assisted PEF ^1^H NMR spectrum (Fig. S6 and S7†), was the absence of a noticeable signal at 6.53 ppm in TCE-d_2_ (residual solvent signal is set at 6.00 ppm). This signal corresponds with a product of an undesired side reaction; the decarboxylation of FDCA. Decarboxylation is undesired as it caps the polymer chain and thus prevents further chain growth.^13^ The absence of the decarboxylation signal indicates that a catalyst free polymerization could be beneficial to suppress the decarboxylation reaction.

### Isosorbide-based polyesters

**Fig. 4:**
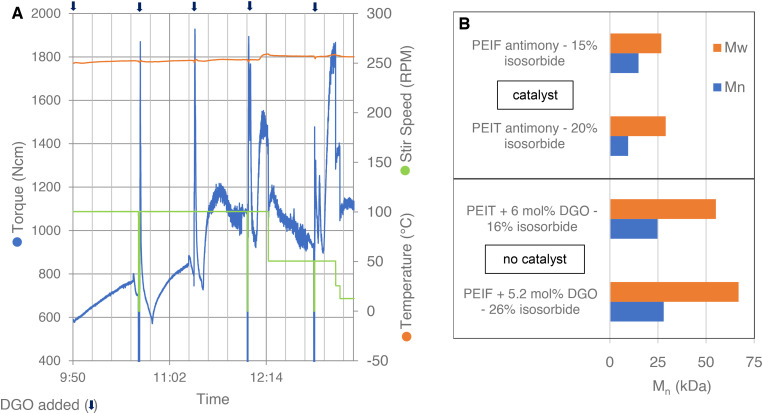
Autoclave polycondensation. (A) Data read-out of the PEIT experiment: torque (blue), temperature (orange) and stir speed (green). The experiment starts after a short polycondensation of PEIT without catalyst. The chain extender (DGO) is added portion wise 5×, in total 6 mol% (1.9, 1.2, 1.2, 0.8, 0.9 mol%); each addition of DGO is marked with an arrow at the top. (B) At the top, molecular weights reported in literature for PEIT and PEIF produced by a traditional polycondensation with antimony catalyst.^27,28^ At the bottom, molecular weight of PEIT and PEIF produced with the molecular weight booster and no catalyst. The reported percentages are in mol and are based on the total FDCA or TPA content. See Fig. S8 and S9† for more detailed results.

Isosorbide, being a secondary diol, is infamously unreactive and therefore it is difficult to obtain high molecular weight polyesters with high isosorbide content.^26–28^ Consequently, the molecular weights obtained for PEIT and PEIF in literature are generally too low for most applications. This is likely one of the reason why there are no publications on the production of PEIF, examples can only be found in patents.^26^ To further explore the molecular weight booster's application and to test its scale-up potential, PEIT and PEIF were produced on a kg-scale in a 2 L autoclave, see Experimental section **[I]** and **[J]** for the synthesizes. Small amounts of DGO were added stepwise to a polycondensate of PEIT or PEIF, without catalyst. Equipped with a torque sensor, the autoclave provides us with valuable insights into the molecular weight, as it is known that the torque increases alongside the molecular weight under similar temperature and stirring speed conditions.^52^ The autoclave data read-out of the DGO-assisted PEIT polymerization is shown in Fig. 4A. DGO was added stepwise to the PEIT polymer mixture. This led to steep increases in torque after each addition of DGO, which clearly demonstrates the impact DGO has on the molecular weight. A comparison of the results obtained with DGO addition to those from traditional polycondensation is shown in Fig. 4B. Even in the absence of Sb catalysts, the molecular weights obtained with the assistance of DGO are much higher. In the case of PEIT the *M*_n_ increases from 9.5 kDa to 24.8 kDa. In spite of the considerably higher isosorbide content in the DGO-assisted PEIF experiment, there is a remarkable enhancement of 13.1 kDa compared to the traditional polycondensation. This demonstrates that with the molecular weight booster, remarkably high molecular weights are easily reached, even without any added catalyst. The *T*_g_ for PEIT (16% IS) was 93 °C and the *T*_g_ for PEIF (26% IS) was 110 °C (Fig. S8†). These findings demonstrate that PEIT and PEIF with a high *T*_g_ can be obtained at molecular weights suitable for a wide range of applications. Additionally, the results indicate that the DGO-assisted polymerization is scalable from small-scale glassware to a larger-scale metal reactor.

### Closer look at observing oxalate in the final polymer

The presence of oxalate in the final polymer can be observed in several ways. One way is that oxalate lowers the *T*_g_ of the material as it is less rigid than terephthalate and furanoate. For PET it is expected that oxalate would decrease the *T*_g_ by about 0.4 °C per mol% (based on total diacid). For example, the *T*_g_ for PETO with 48% oxalate is 58 °C and poly ethylene oxalate is 36 °C.^48^ As furanoate is slightly more rigid than terephthalate, the presence of oxalate in PEF is expected to lower the *T*_g_ somewhat more pronounced. If we now look at the thermal properties of the DGO assisted PET, no unexpected lower *T*_g_ values compared to non-boosted PET were observed, see Table 1. This indicates that the oxalate is removed from the final polymer under the used polymerization conditions. However, it should be kept in mind that several other factors also influence the *T*_g_, such as side products that act as monomer (diethylene glycol) or the molecular weight. This makes it difficult to make a concrete conclusion solely on *T*_g_, especially for smaller oxalate contents.

**Table 1:** Effect of oxalate on the thermal properties of the polymer. The mentioned percentages are based on the total of diacids (terephthalate + oxalate). No catalyst were added for the polymerizations. See experimental section [B], [D] and [E] for the synthesis details

	Feed	NMR	GPC	GPC	GPC	DSC	DSC
Type:	DGO (mol%)	DEG content (mol%)	*M*_n_ (kDa)	*M*_w_ (kDa)	PDI (*M*_w_/*M*_n_)	*T*_g_ (°C)	*T*_m_ (°C)
PETO-48%	49	1.7	17.9	35.6	1.99	57.9	184.6
PET – non boosted	—	4.5	16.4	33.4	2.03	77.2	253.3
PET – boosted	2.2	4.2	27.1	54.6	2.02	78.1	251.2

### Detecting oxalate by ^13^C NMR

**Fig. 5:**
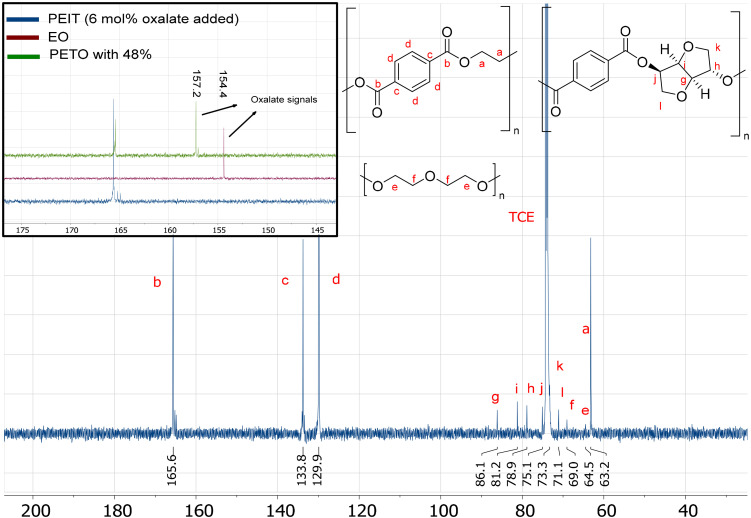
^13^C NMR of the molecular weight boosted PEIT (6.0 mol% DGO added). The anticipated region where oxalate would appear is emphasized by overlaying the ^13^C NMR spectra of PETO and ethylene oxalate; 157.2 ppm for oxalate in the polymer and 154.4 ppm for ethylene oxalate. No oxalate signals could be observed in the PEIT spectra where 6 mol% of DGO was added. Note that, PETO and PEIT are dissolved in TCE-d_2_ and EO was dissolved in DMSO-d_6_ (due to low solubility in TCE). The mentioned percentages are based on the total of diacids (terephthalate + oxalate).

Another way to detect oxalate is by NMR spectroscopy. ^13^C NMR can be used to detect oxalate directly, for this the breadth of the ^13^C spectrum helps to specifically identify oxalate and the two similar carbons of oxalate help with heightening the sensitivity. Using the previously synthesized PETO and EO, it was determined that oxalate within the polymer can be detected at 157.2 ppm and EO at 154.4 ppm. For the PEIT produced *via* the molecular weight boosting strategy (6 mol% oxalate added), no signals of oxalate can be seen in the ^13^C NMR spectrum (Fig. 5): the elevated temperatures in combination with the vacuum caused the removal of all oxalate as EO. However, it is important to note that ^13^C NMR has a relative low sensitivity making it more difficult to detect low concentrations. The 3.3 mol% of DEG was still detectable,^53^ which means oxalate should be detectable up to at least this concentration, assuming similar relaxation time.

### Detecting oxalate by ^1^H NMR

**Fig. 6:**
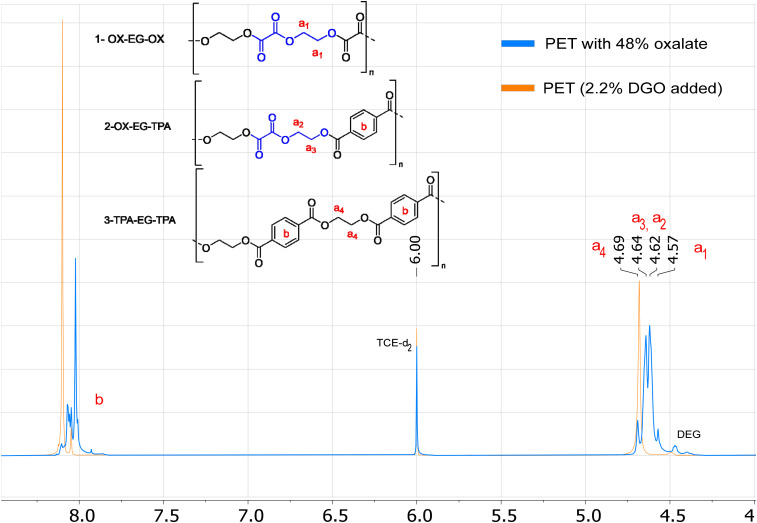
Superimposed ^1^H NMR spectra of the molecular weight boosted PET (2.2 mol%) and PETO with 48% oxalate. The blue spectrum represents the NMR spectrum of PETO with 48% oxalate and the orange spectra that of the PET produced by assistance of 2.2 mol% DGO. The chemical shifts of the different ethylene glycol oxalate and terephthalate esters are highlighted. The mentioned percentages are based on the total of diacids (terephthalate + oxalate).

The most signal sensitive and easy method for detection of oxalate was by ^1^H NMR. Oxalate can be detected *via* the (di)ethylene glycol oxalate ester, as the protons of the ethylene glycol oxalate ester shift upfield (4.64–4.57 ppm) *versus* ethylene glycol terephthalate esters (4.69 ppm), see Fig. 6. Observing the six-membered EO ring is more challenging due to its similar chemical shift to that of the ethylene glycol terephthalate esters, specifically at 4.68 ppm (see Fig. S3†). However, by analyzing the ratio between the integrals of the acids and diols, allows us to infer the presence of both EO and oxalate esters. When oxalate is present the integral ratio between acids (terephthalate or furanoate) and diols (EG + DEG + ISO) increases above 1.00 : 1.00. For example, PETO-48% has a ratio of 1.00 : 1.92 and PET has a ratio of 1.00 : 1.00. In this method, it is assumed that all free acid has reacted to form esters, which is actually necessary for obtaining high molecular weight polyesters. Only for the end groups it is possible not to have undergone esterification. Given the excess diol conditions employed in our polymerizations, we can safely assume that the majority of end groups has undergone esterification. Even in the extreme scenario that all end groups are still acid end groups, this would give <2 mol% end groups for a polymer with *M*_n_ above 20 kDa (PET repeating unit: 192.2 g mol^−1^), which is equal to <1 mol% uncertainty. Properties remain unaffected when the oxalate content falls below these levels, rendering the DGO addition virtually traceless. If we now look at ^1^H NMR of the DGO-assisted PET and non DGO-assisted PET (Fig. S10 to S12†), the TPA : diol ratios are similar 1.00 : 1.00, instead of the 1.00 : 1.02 when all oxalate would not have been removed. Additionally, no specific signals of oxalate can be seen at 4.64 to 4.57 ppm. The removal of oxalate is even more evident in the PEIT and PEIF samples, where a more significant amount of oxalate was added (5–6 mol%), no oxalate signals were detected in the ^1^H NMR spectra. (Fig. S13 and S14†).

### Purpose EO and guaiacol

An important aspect to consider from the DGO-assisted polymerization is the fate of the EO and guaiacol formed as side products. Even though relatively small amounts of DGO are used, at a ton scale polymer production it would result in kgs of guaiacol and ethylene oxalate. To make the process more economic and sustainable, the condensate should have a designated use. Guaiacol can be reused in the DGO synthesis process. Ethylene oxalate could likely be reused for DGO synthesis as well, however it is probably more efficient to use it directly as a monomer. It could for example be used to produce poly ethylene oxalate by ring opening polymerization,^48^ or it could be combined with other ring opening monomers such as lactide and glycolide to produce copolymers of PLA and PGA. Here, oxalate could for example reduce crystallinity, provide better physical properties or improve biodegradability.^47^

### 3D printing

**Fig. 7:**
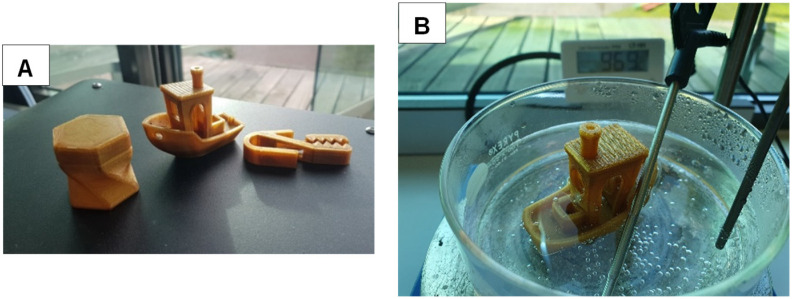
Pictures of metal free PET and PEIT. (A) From left to right 3D printed storage box, benchy and clamp (B) benchy submerged in hot water (97 °C).

350 g of PEIT (16% isosorbide) was successfully processed into filament for 3D printing (*∅* 2.85 mm). The PEIT filament was strong enough to be printable with a molar mass of 14.7 kDa (Fig. S9†). Several 3D printing designs were made from this filament (Fig. 7). Submerging the material in 97 °C water confirmed its resistance to heat.

## Conclusions

This research has shown that DGO can be effectively used as molecular weight booster to generate high molecular weight PET, PEF, PEIT and PEIF polyesters. Compared to commonly used chain extenders, DGO has the advantage that it can be removed from the polymer product. This means that apart from increasing the molecular weight, it has no influence on the polymer's properties. When synthesizing polyesters, applying DGO therefore provides increased molecular weight without the inherent drawbacks of commonly used chain extenders.

For PEF, molecular weights were produced close to what is normally obtained after a 24-hour solid-state polymerization. For amorphous polyesters SSP is not even an option, which currently limits the maximum *T*_g_ at which PEIT can be produced and addition of DGO to this process actually opens up a pathway to industrially produce such materials. As long as the polyester contains sufficient amounts of ethylene glycol monomers, DGO should work.

DGO is very reactive, which opens the door to the production of polyesters such as PET and PEF without catalyst or with more benign, but less effective catalysts, even when a large amount of unreactive isosorbide is incorporated. This is of particular relevance due to the health risks associated with antimony, which is the preferred catalyst for PET production.^18^ Consequently, the leaching of this element from PET into packaged products and the environment poses a significant concern.^14–17^ Moreover, due to the utilization of antimony at very low concentrations, effectively recovering it from waste plastic becomes virtually impossible.^19,54–56^ Also the future availability of Sb is an important issue, as reserves are running low. Furthermore DGO presents a workable alternative to the commonly used solid state polymerization, saving on time and energy. The simplicity of this method makes it scalable and production of the booster itself can also be scaled, as DGO can be effectively synthesized by the transesterification of dimethyl oxalate with guaiacol. Unlike the metal catalysts used for polymerization, the catalyst used for the production of DGO can be easily recovered and recycled.^57^ Guaiacol is also non-toxic and has the potential to be derived from lignin, an abundant renewable resource.^58^

DGO's high reactivity and easy removal from the polymer could also help solve current issues in mechanical recycling of polyesters. This is especially relevant for PET, due to its production scale, where the decrease in molecular weight due to thermal degradation with every recycle and the accompanying loss in quality severely limits closed-loop recycling. The addition of small amounts of DGO to such a melt can readily boost the molecular weight back to sufficient levels, making the recycled PET fit for purpose.

## Experimental section

### Materials

Ethylene glycol (EG; >99%), titanium(iv) butoxide (97%) and tetraethylammonium hydroxide solution (TEAOH; 35 wt% in H_2_O) were supplied by Sigma Aldrich. Terephthalic acid (TPA; >99%) was supplied by Fisher Scientific. Guaiacol (99%) was purchased from Carbosynth. Isosorbide (>99.5%) was supplied by Roquette. Dimethyl oxalate (DMO; >99%) and bis(2-hydroxyethyl) terephthalate (BHET; >85%) were bought from TCI chemicals. Tetrahydrofuran (THF; 99%) and diethyl ether (99%) were supplied by VWR International. 1,1,2,2-Tetrachloroethane-d_2_ (TCE-d_2_; 99.5%) and dimethyl sulfoxide-d_6_ (DMSO-d_6_; 99.8%) were ordered from ABCR chemicals. 2,5-Furandicarboxylic acid (>99%) was kindly provided by Avantium. Titanium tetra(phenolate) was produced by a described patent procedure.^57^ Diguaiacyl oxalate was synthesized (see below).

### Characterization

**NMR –** ^1^H-NMR and ^13^C-NMR spectra were recorded at appropriate frequencies on a Bruker AMX 400 (^1^H, 400.13 MHz) and Bruker DRX 500 (^1^H, 499.91 MHz) spectrometers. Chemicals shift are referenced to residual proton in the specified solvent.

^1^H NMR sample preparation: ∼8 mg of polymer was dissolved in 0.6 mL TCE-d_2_.

^13^C NMR sample preparation: A very high concentration PEF sample (50 mg mL^−1^) was made at the reflux temperature of TCE-d_2_ (150 °C). The ^13^C-NMR was measured in 3 sessions of 1024 scans (3 × 1 hour). Between each measurement, the sample was heated to make sure the PEF did not precipitate out of solution. The PEIF sample was recorded overnight (50 mg mL^−1^) with a total of 22132 scans and did not needed to be heated in between.

**DSC –** Differential scanning calorimetry thermograms were obtained with a Mettler Toledo DSC 3 STAR^e^ system. Around 5 mg of sample was weighed in a standard aluminum crucible (40 μl). Next, the sample was analyzed in three steps, under a nitrogen flow of 50 ml min^−1^. First, after stabilizing at 20 °C for 5 minutes, the sample was analyzed at a rate of 10 °C min^−1^ from 20 to 230–250 °C. Second, the sample was cooled down to the starting temperature of 20 °C with a cooling rate of 50 °C min^−1^. Lastly, the first step is repeated, and the data of this cycle is used for reporting.

For PET, the sample was heated to 300 °C with 10 °C min^−1^, then cooled at the maximum rate by setting the temperature at 25 °C for 5 minutes. Subsequently, heated again from 25 to 300 °C with 10 °C min^−1^, the values from this cycle are reported.

**SEC –** For *PEIF* – Molecular mass distributions were measured using size exclusion chromatography (SEC) on a Shimadzu LC-20AD system with two PLgel 5 μm MIXED-C columns (Polymer Laboratories) in series and a Shimadzu RID-10A refractive index detector, using dichloromethane as mobile phase at 1 mL min^−1^ and *T* = 35 °C. The molecular weights were calculated based on polystyrene standards.

For *PET*, *PEF and PEIT* – SEC measurements were carried out on a Hitachi Chromaster 5450 with a Agilent HPLC system equipped with two PFG 7 micrometer (μm) Linear M (300 × 7.5 mm) columns. HFIP was used at mobile phase with a 1 mL min^−1^ flow and *T* = 35 °C. Refractive index detector (Chromaster 5450) and Intrisc viscosity meter (Viscostar) was used for analysis. ASTRA 6.1 (Wyatt technology) software was used for further processing. The molecular weights were calculated based on PMMA standards.

### Filament making

*PEIT*: The filament was made on a Precision 350 filament maker from the company 3Devo. The following settings were used: heater 1 (240 °C), 2 (225 °C), 3 (220 °C), 4 (210 °C), screw speed (5 RPM), fan speed 5% and filament diameter 2.85 mm.

### 3D printing

The 3D models were printed on a Ultimaker 3 Extended. The heat plate was prepared by coating with a glue stick. The following settings were used for PEIT: Heat plate (85 °C), Nozzle (230 °C), infill (20–100%), print speed (60 mm s^−1^), fan speed (20%) and layer height (0.2 mm).

### General protocol solid state polymerization

The resin obtained after melt polymerization was grinded and sieved, after which the sieve fraction of 0.6–2.0 mm was dried/crystallized overnight in an oven at a temperature of 150 °C. The material obtained was subjected to solid state polymerization for 24 h under nitrogen atmosphere at an oil temperature of 200 °C. After cooling to room temperature, the resin obtained was sieved, after which the sieve fraction of 1.4–2.0 mm was subjected to further analyses.

### Injection molding

Tensile bars were obtained with a Thermo Scientific HAAKE Minijet II apparatus equipped with an ISO-527-2-A5 mold. The pressure was set at 1000 bar, mold temperature at 15 °C and the pressure time was set at 6 seconds. The cylinder temperature was set at 280 °C. When temperature and pressure was stable, the cylinder was checked to make sure no residues from previous runs remained. Before each press, the mold was coated with Teflon mold release spray, and when dry, placed in the holder. Next, 2.2–2.7 grams of polymer was weighed and transferred inside the cylinder. The polymer was left to melt for 60 seconds inside the cylinder. Subsequently, the cylinder was placed on top of the mold, the door was closed, and the injection program was started. After the injection program, the mold was taken apart and the sample was removed and examined for defects. The excess polymer in the cylinder was discharged before each injection run. A typical tensile bar (ISO-527-2-A5) weighed 1.8 gram.

### Tensile testing

The tensile bars were analyzed on an Instron 5565 machine with load cell (1 kN) and Instron strain gauge extensometer 2630–106 (25 mm). Sample size were set to width (4 mm), thickness (1.95 mm) and parallel length (25 mm). The tensile tests were performed at a test speed of 5 mm s^−1^ and speed was increased to 50 mm s^−1^ after 0.5 mm mm^−1^ strain was reached. When the maximum elongation of the extensometer was reached (25 mm) the extension of the frame was used to determine the elongation at break.

### Inductively coupled plasma metal analysis

DGO, FDCA, BHET and PEF oligomers were analyzed by ICP analysis for common metals used in polymerization chemistry. PEF oligomers represent the polycondensation polymer before adding DGO. None of these metals were detected in significant amounts. See ESI† for the data overview.

**Sample preparation:** 500 mg product was dissolved in 5 mL HNO_3_, for BHET 150 mg was used. Acid digestion was performed using an Anton Paar Microwave digestion system. After digestion, 5 mL MiliQ was added to a total of 10 mL and filtered. Next, 100 μL of sample together with 50 μL of 1000 ppm yttrium was added to a total of 10 mL HNO_3_ solution (1% (v/v%) in MilliQ). Yttrium served as internal standard.

Analysis: Inductively Coupled Plasma (ICP) analysis was performed using a PerkinElmer Avio200 equipped with Optical Emission Spectrometer (OES). Samples were taking using an auto sampler. The system was calibrated using 0, 2.5, 5, and 7.5 ppm standards in HNO_3_ solution (1% (v/v%) in MilliQ) for the following elements using 5 ppm yttrium as an internal standard.

### Synthesis

#### [A] 2L autoclave synthesis of DGO from DMO and guaiacol


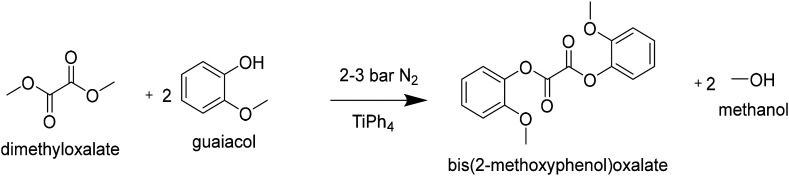
1192 g guaiacol (9.6 mol; 2.94 eq.), 386 g dimethyloxalate (3.27 mmol; 1 eq.) and 2.9 g titanium tetra(phenolate) (5 mmol; 1.7 meq) were transferred to a steel 2 L kiloclave (Buchi). The reactor pressure was set to 3 bar with a N_2_ bleed of 2 L h^−1^. The heater oil temperature was set to 275 °C (245 °C internal), when the reactor temperature reached 100 °C the stirrer was started and gradually increased to 100 rpm. The reaction was followed by removing and monitoring the products in the condensation flask. After 4 hours the pressure was gradually lowered to 2 bar, and the reaction was continued for 2 hours. Subsequently, the oil temperature was set to 250 °C (225 °C internal) and the pressure was gradually lowered to atmospheric pressure to facilitate the distillation of guaiacol. When distillation came to a halt, the pressure was lowered further by using a vacuum pump. When a pressure of 10 mbar was reached, the condensation flask was drained to make room for the DGO product. Next, full vacuum was applied (<0.1 mbar) at an oil temperature of 260 °C to facilitate the distillation of the DGO product into the condensation flask. The condensation product (300 g; 30% yield) was dissolved in THF (0.5 L), and left to crystallize overnight. The obtained crystals were filtered off and washed twice with (0.2 L) diethyl ether. The crystals were dried at 60 °C under vacuum (1 mbar). The obtained product (225 g, 22.5% yield) was analyzed by ^1^H-NMR and DSC. The product had a melting point of 126.2 °C (>99 mol% purity by DSC).

For the ^1^H NMR spectrum and DSC curve see ESI Overview S4.†

#### [B] Small scale synthesis of PETO-48%

**Pre-drying BHET** – 30.00 g of BHET (118 mmol; 1 eq.) was weighed in a 100 mL 3 neck round-bottom flask. The round-bottom flask was put in an oil bath and was equipped with a mechanical stirrer, nitrogen inlet and short path connected Schlenk flask, all suited for high vacuum. Nitrogen flow was set to 50 mL min^−1^ and temperature of the oil bath was set to 140 °C. The set temperature was reached in about 15 minutes. As soon as a melt was observed, the stirring speed was set to 100 RPM. The temperature was kept at 140 °C for 1 hour. **Polymerization** – The oil temperature was set to 175 °C and 35.134 g of DGO (116.2 mmol; 0.985 eq.) was added to the BHET. After 1 hour, vacuum was applied and the temperature was set to 190 °C. The pressure was reduced until guaiacol slowly came over (∼125 mbar) and was halved every 15 minutes. When 10 mbar was reached, the temperature was increased to 210 °C. After 2 hours, a complete vacuum of <1 mbar was applied and sustained while stirring for 1 hour. Subsequently, the vacuum was released using nitrogen (N_2_), and the molten resin was taken out of the reactor.

#### [C] Small scale synthesis of PETO-19%

**Oligomer synthesis** – 20.00 g of BHET (78.7 mmol; 0.68 eq.), 6.2731 g of TPA (37.8 mmol; 0.32 eq.) and 4 g of guaiacol (32.2 mmol; 0.28 eq.) was weighed in a 100 mL round-bottom three-neck flask. The round bottom flask was put in an oil bath and was equipped with a mechanical stirrer, nitrogen inlet and short path connected Schlenk flask, all suited for high vacuum. Nitrogen flow set to 50 mL min^−1^ and temperature of the oil bath was set to 238 °C. The set temperature was reached in about 20 minutes. As soon as a melt was observed, the stirring speed was set to 100 rpm. After 2.5 hours, a clear melt was observed.

**Polymerization –** An hour after a clear melt was observed, 10.0 g DGO (33.1 mmol; 0.28 eq.) was added to the reaction flask. The pressure was reduced until guaiacol slowly came over (∼300 mbar). Next, the pressure was halved each 15 minutes. After 2 hours full vacuum was applied (∼1 mbar), and was maintained while stirring for 1 hour. Vacuum was then replaced by a nitrogen flow and the polymer was taken out of the flask. This indicates that around 400 mg of EG (6.3 mmol; 0.05 eq.) gets removed in the oligomer synthesis.

#### [D] Preparation of the PET-oligomers for the PET synthesizes

A 250 mL three neck round bottom flask was charged with 51.22 g bis(2-hydroxyethyl) terephthalate (0.20 mol, 1 eq.) and 30.27 g terephthalic acid (0.18 mol, 0.9 eq.). The round bottom flask was put in an oil bath and equipped with a mechanical stirrer, nitrogen inlet and short path connected Schlenk flask, all suited for high vacuum. The polymerization was carried out in three steps, esterification, polycondensation and molecular weight boosting step.

In the esterification step, nitrogen flow was set to 50 mL min^−1^, stir speed to 100 rpm and the temperature of the oil bath was set to 250 °C. The set temperature was reached in 35 minutes and maintained for 3 hours. Subsequently, the temperature was increased to 270 °C, 1 hour later a clear melt was obtained and the esterification was continued for another 1.5 hours. After this, the stirrer was stopped, nitrogen flow was increased to 100 mL min^−1^ and with a spatula 64 g of white crystalline PET-oligomers was taken out of the reactor. These oligomers were then used to produce PET without catalyst.

#### [E] DGO-assisted PET with no catalyst

In a similar setup, but now 100 mL three neck round bottom flask, 23.097 g (0.120 mol, 1 eq.) of the previously made PET-oligomers were charged. Nitrogen flow was set to 50 mL min^−1^, stir speed to 100 rpm (as soon it was melted) and the temperature of the oil bath was set to 280 °C. The set temperature was reached in 40 minutes and was maintained for the complete polymerization. After 2.5 hours, polycondensation was started by applying vacuum. The vacuum was reduced by half every 5 minutes, starting from 400 mbar, after 40 minutes full vacuum applied <1 mbar and maintained for 30 minutes. After this, 0.783 g (0.003 mol, 0.022 eq.) of bis(2-methoxyphenol) oxalate was added to the round bottom flask by replacing the vacuum with a nitrogen flow. Subsequently, full vacuum was applied for an additional 1.5 hours. After this, the stirrer was stopped, nitrogen flow was increased 100 mL min^−1^ and with a spatula the polymer was taken out of the flask.

**PET no catalyst and without the booster –** For the PET without the molecular weight booster, a similar procedure was used: 19.84 g (0.103 mol) of PET-oligomers was charged, no booster was added, but the polycondensation time was longer: 3.5 hours instead of 1.5 hours.

#### [F] Small scale DGO assisted polymerization of PET in the presence of a metal catalyst

A 100 mL three neck round bottom flask was charged with 33.559 g bis(2-hydroxyethyl) terephthalate (132 mmol, 1 eq.) and 0.022 g titanium(iv) butoxide (0.07 mmol, 0.5 meq.). The round bottom flask was put in an oil bath and equipped with a mechanical stirrer, nitrogen inlet and short path connected Schlenk flask, all suited for high vacuum. The polymerization was carried out in three steps, esterification, polycondensation and molecular weight boosting step.

In the esterification step, nitrogen flow was set to 50 mL min^−1^, stir speed to 100 rpm and the temperature of the oil bath was set to 250 °C. The set temperature was reached in about 40 minutes and esterification was conducted for 2 h. The oil bath temperature was set to 270 °C and vacuum was applied to the reactor (400 mbar). The pressure was slowly decreased to <1 mbar within one hour. The melt was stirred at <1 mbar for 20 minutes, after which the stirring speed was decreased to 30 rpm and the oil bath temperature was increased to 300 °C. After 20 minutes the oil bath temperature reached 300 °C and the reactor was purged with nitrogen. A melt sample was taken from the reaction mixture and 0.2 g DGO (0.66 mmol, 0.5 mol%) were added. The reaction mixture was stirred for 5 minutes under a nitrogen atmosphere. Then a vacuum of <1 mbar was applied and the reaction mixture was stirred for 20 minutes. The reactor was purged with nitrogen and the polymer was removed from the reactor.

#### [G] Small scale typical PEF polymerization from FDCA in presence of metal catalyst

A mixture of 2,5-furandicarboxylic acid (30 g, 19.2 mmol), Sb_2_O_3_ (10.5 mg, 0.036 mmol), TEAOH (21 mg 35wt% water solution, 0.050 mmol) and ethylene glycol (14.30 g, 23.0 mmol) was stirred under N_2_ atmosphere and heated to an oil temperature of 200 °C, and after 20 minutes the oil temperature was raised to 220 °C. After keeping the oil temperature at 220 °C for 180 minutes, vacuum was applied to lower the pressure from 1000 mbar to 1 mbar in *ca.* 20 minutes. As soon as the pressure reached 100 mbar, the oil temperature was raised to 260 °C. Polycondensation (at *P* ≤ 1mbar and *T*_oil_ = 260 °C) was conducted for 75 minutes, after which vacuum was released with N_2_ and the melt resin was taken out of the reactor.

#### [H] Small scale DGO assisted polymerization of PEF in absence of metal catalyst

A 100 mL three neck round bottom flask was charged with 20.6 g 2,5-furandicarboxylic acid (132 mmol; 1 eq.), 10.3 g ethylene glycol (166 mmol; 1.25 eq.) and TEAOH (15 mg 35wt% water solution, 0.036 mmol). The round bottom flask was put in an oil bath and equipped with a mechanical stirrer, nitrogen inlet and short path connected Schlenk flask, all suited for high vacuum. The polymerization was carried out in three steps, esterification, polycondensation and molecular weight boosting.

In the esterification step, nitrogen flow was set to 50 mL min^−1^ stir speed 100 rpm and the temperature of the oil bath was set to 220 °C. The set temperature was reached in about 30 minutes. The first esterification step was considered complete after a clear melt was obtained, which was obtained after 2 hours. The esterification was continued for an additional 30 minutes before polycondensation was started.

In the polycondensation step, temperature was set to 260 °C and vacuum was applied (200 mbar). Vacuum was slowly decreased to <1 mbar in 30 minutes. Full vacuum was maintained for 1 hour before starting with the molecular weight boosting.

In the boosting step, under a nitrogen flow, DGO was added to the melt. After the addition, the melt was stirred for 5 minutes after which vacuum was slowly applied.

**For the 3.5 mol% stepwise experiment:** In total 3 additions of DGO were done, first two additions of 0.4 g (1 mol%) DGO and a final addition of 0.6 g DGO (1.5 mol%). Between each addition it took 15 minutes until full vacuum was reached and was maintained for 30 minutes until the next addition.

#### [I] 2L autoclave PEIT polymerization from BHET in absence of metal catalyst

503.7 g BHET (1.98 mol; 1 eq.), 82.1 g isosorbide (0.56 mol; 0.284 eq.) and 33.2 g terephthalic acid (0.199 mol; 0.1 eq.) were charged to a steel 2 L kiloclave (Buchi). The oil temperature was set to 250 °C with a N_2_ bleed of 2 L h^−1^. The set temperature was reached in about 20 minutes (internal ∼230 °C). As soon as the internal temperature reached 200 °C, stir speed was set to 100 rpm (anchor stirrer). After 3 hours of esterification the nitrogen flow was halted, vacuum was applied and the oil temperature was set to 270 °C (250 °C internal). In about 1 hour full vacuum was reached (<1 mbar), and maintained for 2 hours. The torque after polycondensation increased from 500 Ncm to 570 Ncm. 130 g of condensate was collected at this stage.

The next day, 12.4 g (1.8 mol%) of DGO was added to the cooled down post polycondensate. Subsequently, oil temperature was set to 270 °C and a N_2_ bleed of 2 L h^−1^ was set. As soon as the internal temperature reached 200 °C, stir speed was set to 100 rpm. After 15 minutes at the set temperature, nitrogen flow was halted, and vacuum was applied. In about 30 minutes the Torque steadily went up to ∼750 Ncm. Then another four times DGO was added 8, 8, 5 and 6.2 gram (total 39.6 g, 6 mol%). After each addition the reaction was stirred 5 minutes under a nitrogen flow, after which vacuum was applied for 30 minutes before the next addition. The final torque reached was 1100 Ncm at 12 RPM. The polymer was extruded in about 1.5 hours by using N_2_ pressure. The extruded polymer was guided through a water bath and was chipped. The final yield was 350 g (70%) of golden brown polymer chips.

Details regarding the autoclave parameter readout throughout this polymerization experiment are available in the ESI: Overview S4† additional experimental results and analysis.

#### [J] 2L autoclave PEIF polymerization from FDCA in absence of metal catalyst

702.0 g 2,5-furandicarboxylic acid (4.50 mol; 1 eq.) 191.0 g isosorbide (1.31 mol; 0.290 eq.), 265.8 g ethylene glycol (4.28 mol; 0.95 eq.) and 0.5 mL TEAOH (35% water solution, 1.2 mmol) were charged to a steel 2 L kiloclave (Buchi). The oil temperature was set to 230 °C with a N_2_ bleed of 2 L h^−1^. The set temperature was reached in about 15 minutes (internal ∼215 °C). As soon as the internal temperature reached 175 °C, stir speed was set to 125 RPM (anchor stirrer). After 3.5 hours of esterification the nitrogen flow was halted. At this stage 188 g of condensate was collected. Next, vacuum was applied and the oil temperature was set to 260–275 °C (243–255 °C internal). In about 30 minutes full vacuum was reached (<1 mbar), and maintained for 2 hours. The torque after polycondensation increased from 500 Ncm to 550 Ncm. The next day, 40 g (3 mol%) of DGO was added to the cooled down post polycondensate. Subsequently, oil temperature was set to 275 °C with a N_2_ bleed of 4 L h^−1^. As soon as the internal temperature reached 220 °C, stir speed was set to 100 RPM. After 15 minutes at the set temperature, nitrogen flow was halted, and vacuum was applied. Subsequently, another three times DGO was added 15, 10 and 6 gram (total 71 g, 5.2 mol%). After each addition the reaction was stirred 5 minutes under a nitrogen flow, after which vacuum was applied for 30 minutes before the next addition. The final torque reached was 1020 Ncm at 12 RPM. The polymer was extruded in about 1.5 hours by using N_2_ pressure. The extruded polymer was guided through a water bath and was chipped. The final yield was 650 g (78%) of dark polymer chips.

Details regarding the autoclave parameter readout throughout this polymerization experiment are available in the ESI: Overview S4† additional experimental results and analysis.

## Author contributions

G. J. M. G., B. W., and K. M. conceived the concept. B. W., K. M. and R. J. P. devised the experimental program. K. M. and D. H. W. performed experiments on polyester synthesis and its property characterization. K. M., R. J. P. and G. J. M. G. wrote the manuscript.

## Abbreviations

BHET: Bis(2-hydroxyethyl) terephthalate
DEG: Diethylene glycol
DGO: Diguaiacyl oxalate
DMO: Dimethyl oxalate
DMSO: Dimethyl sulfoxide
DSC: Differential scanning calorimetry
EG: Ethylene glycol
EO: Ethylene oxalate
FDCA: Furan-2,5-dicarboxylic acid
HFIP: Hexafluoroisopropanol
HNO_3_: Nitric acid
HPLC: High pressure liquid chromotography
ICP: Inductively coupled plasma
IV: Intrinsic viscosity
kDa: Kilodalton
*M*_n_
: Number average molecular weight
MPa: Megapascal
*M*_w_
: Mass average molecular weight
Ncm: Newton centimeter
OES: Optical emission spectrometer
PDI: Polydispersity index
PEF: Polyethylene furanoate
PEIF: Polyethylene-*co*-isosorbide furanoate
PEIT: Polyethylene-*co*-isosorbide terephthalate
PET: Polyethylene terephthalate
PETO: Polyethylene terephthalate-*co*-oxalate
Poly: Polymer
RPM: Rounds per minute
PGA: Poly glycolic acid
PLA: Poly lactic acid
SEC: Size exclusion chromatography
SSP: Solid-state polymerization
TCE: Tetrachloroethane
TEAOH: Tetraethylammonium hydroxide
*T*_g_
: Glass transition temperature
TGA: Thermogravimetric analysis
THF: Tetrahydrofuran
*T*_m_
: Melting point
TPA: Terephthalic acid

## Data availability

Data generated during the current study is available from the corresponding author on request.

Data supporting this article has been included as part of the ESI.†

## Conflicts of interest

The authors of this work declare no competing interests.

## Supplementary Material

GC-026-D4GC02791D-s001
