# Supplementary material for: Catalyst free PET and PEF polyesters using a new traceless oxalate chain extender
Source: Green Chem. 2024 Aug 16;26(22):11182–95. doi: 10.1039/d4gc02791d (PMC11328508; doi:10.1039/d4gc02791d)
Supplement: GC-026-D4GC02791D-s001 [file GC-026-D4GC02791D-s001.pdf]

Supplementary Information (SI) for Green Chemistry.
This journal is © The Royal Society of Chemistry 2024

Supplementary information

Catalyst Free PET and PEF Polyesters Using a New Traceless Oxalate Chain Extender

Kevin van der Maas[1], Daniel H. Weinland[1], Robert-Jan van Putten[1,2], Bing Wang[2], Gert-Jan M. Gruter[1,2]*.

[1] Van't Hoff Institute of Molecular Sciences, University of Amsterdam; Science Park 904 1098 XH Amsterdam, The Netherlands.
[2] Avantium Chemicals BV; Zekeringstraat 29, 1014BV Amsterdam, The Netherlands.
*Corresponding author: "Gert-Jan Gruter" <G.J.M.Gruter@uva.nl>

Overview S1 | Reaction mechanisms of DGO with the polymer chain and ethylene oxalate formation.
When DGO is added to PET with ethylene glycol end groups present. First the transesterification reaction between ethylene glycol end group and DGO takes place, releasing one guaiacol:

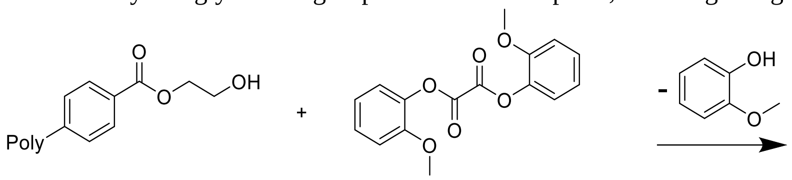

Next, the second transesterification reaction between ethylene glycol end group and DGO end group takes place, releasing another guaiacol:

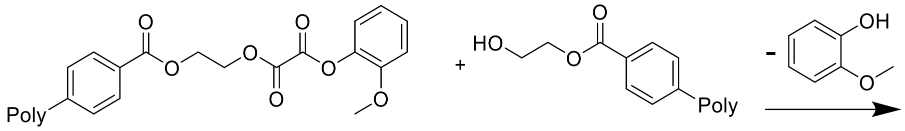

Now, the polymer chains are connected and the chain length is increased:

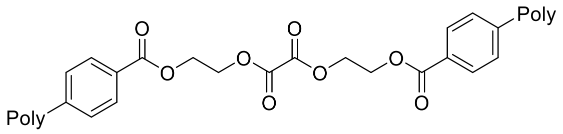

The oxalate can change position within the polymer chain by transesterification, resulting in different repeating structures: TPA-EG-TPA, TPA-EG-OX or OX-EG-OX:

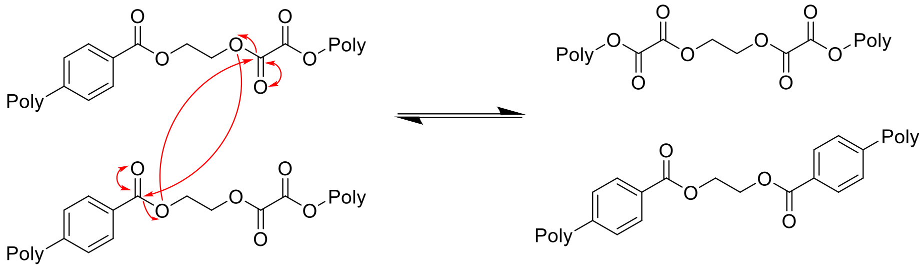

These structures can then undergo internal ring formation to form the six membered ring component ethylene oxalate:

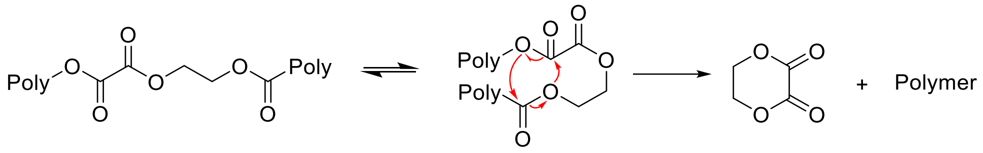

Overview S2 | ICP analysis limit of detection and calibrated wavelength of the measured metals

Element	Abbreviation	Calibrated wavelength (nm)	Limit of detection (LOD) (ppm)
Yttrium	**Y**	371.029	0.5
Tin	**Sn**	189.927	0.2
Molybdenum	**Mo**	202.031	0.2
Zinc	**Zn**	206.2	0.05
Germanium	**Ge**	209.426	0.2
Antimony	**Sb**	217.582	0.2
Lead	**Pb**	220.353	0.2
Cobalt	**Co**	228.616	0.05
Nickel	**Ni**	231.604	0.1
Iron	**Fe**	238.204	0.05
Manganese	**Mn**	257.61	0.05
Chromium	**Cr**	267.716	0.05
Titanium	**Ti**	334.94	0.2

ICP analysis results with an overview of the metals analyzed. (n.d.) = not determined

Results in ppm (weight)	DF	Ar 420.069 (IS) (%)	Y 371.029 (mg/L)	Sn 189.927 (mg/L)	Mo 202.031 (mg/L)	Zn 206.2 (mg/L)	Ge 209.426 (mg/L)	Sb 217.582 (mg/L)
**Lower limit of quantification**			**0.5**	**0.2**	**0.2**	**0.05**	**0.2**	**0.2**
**Sample Blank correction**		**101.87**		**102.87**	**103.87**	**104.87**	**105.87**	**106.87**
PEF oligomers	100	101.85	0.26	<0.2	<0.2	<0.05	<0.2	<0.2
DGO	100	101.69	0.37	<0.2	<0.2	<0.05	<0.2	<0.2
FDCA	100	103.99	0.31	<0.2	<0.2	<0.05	<0.2	<0.2
BHET	68.9	101.93	<1	n.d.	n.d.	<2	<9	<9

Results in ppm (weight)	DF	Pb 220.353 (mg/L)	Co 228.616 (mg/L)	Ni 231.604 (mg/L)	Fe 238.204 (mg/L)	Mn 257.61 (mg/L)	Cr 267.716 (mg/L)	Ti 334.94 (mg/L)
**Lower limit of quantification**		0.2	0.05	0.1	0.05	0.05	0.05	0.2
**Sample Blank correction**		107.87	108.87	109.87	110.87	111.87	112.87	113.87
PEF oligomers	100	<0.2	<0.05	<0.1	<0.05	<0.05	<0.05	<0.2
DGO	100	<0.2	<0.05	<0.1	<0.05	<0.05	<0.05	<0.2
FDCA	100	<0.2	<0.05	<0.1	<0.05	<0.05	<0.05	<0.2
BHET	68.9	<9	<2	n.d.	<2	n.d.	n.d.	<9

Overview S3 | Physical properties of the PET (without catalyst) produced by boosting and without boosting. Additionally, the physical properties of a commercial PET with catalyst and SSP (RAMA PET N180) is given. The raw data can be found below. See experimental section [D] and [E] for the synthesis details.

	Feed	NMR	GPC	GPC	GPC	DSC	DSC	DSC	Polycondensation time (h)
Type:	DGO (mol%)	DEG content (mol%)[a]	M_n (kDa)	M_w (kDa)	PDI (M_w/M_n)	T_g (°C)	T_{cc} (°C)	T_m (°C)	
Non boosted	-	4.5	16.4	33.4	2.033	77.2	126.2	253.3	3.5
Boosted	2.2	4.2	27.1	54.6	2.016	78.1	133.2	251.2	1.5
RAMA PET[b]	-	3.6	36.8	74.5	2.03	78.7	152.8	249.4	SSP

a. The BHET used for polymer synthesis contains 1.3 mol% DEG (based on total TPA), this results in 0.7 mol% DEG from BHET in the final polymer.
b. The commercial Rama PET also contains a portion of isophthalic acid (4%), which should be kept in mind when comparing them to the synthesized polymers.

	**Tensile Modulus**	**Maximum Tensile stress**	**Tensile stress at Yield**	**Extension at Break**
	**(MPa)**	**(MPa)**	**Offset 0.002 mm/mm (MPa)**	**(%)**
Non boosted	2522 ± 169	50.2 ± 11.1	40.6 ± 1.9	5.5% ± 2.0
Boosted (2.2 mol% DGO)	2220 ± 59	57.8 ± 6.7	43.4 ± 7.8	445.8% ± 169.5
RAMA PET	2622 ± 328	65.8 ± 2.3	43 ± 7.6	499.2% ± 91.7

Raw data tensile testing data of PET produced without a catalyst:

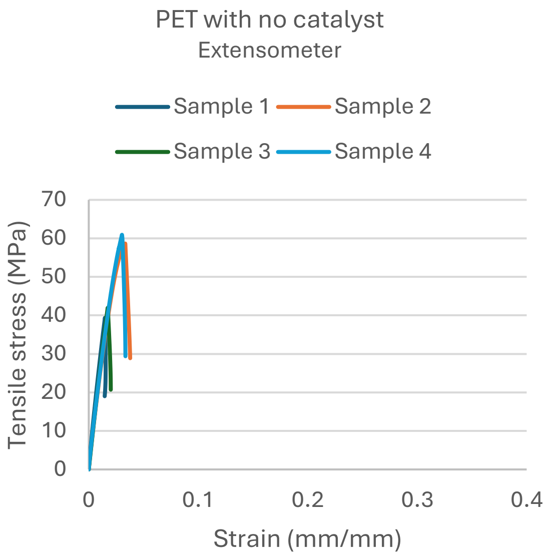

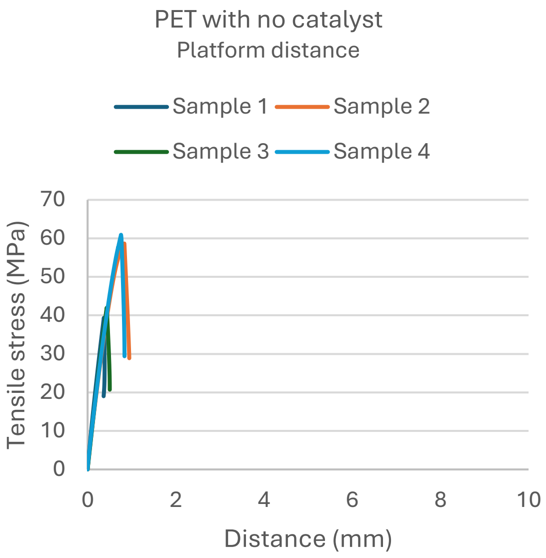

	Tensile Modulus	Maximum Tensile stress	Tensile stress at Yield	Extension at Break (Standard)
	(MPa)	(MPa)	Offset 0.002 mm/mm (MPa)	(%)
Sample 1	2785	39.4	38.1	3.7%
Sample 2	2374	58.7	41.2	7.1%
Sample 3	2556	42.0	40.7	4.0%
Sample 4	2372	60.9	42.6	7.5%
Maximum	2785	60.9	42.6	7.5%
Mean	2522	50.2	40.6	5.5%
Minimum	2372	39.4	38.1	3.7%
Stdev.	169	11.1	1.9	2.0%

Raw data tensile testing data of DGO-assisted PET produced without a catalyst:

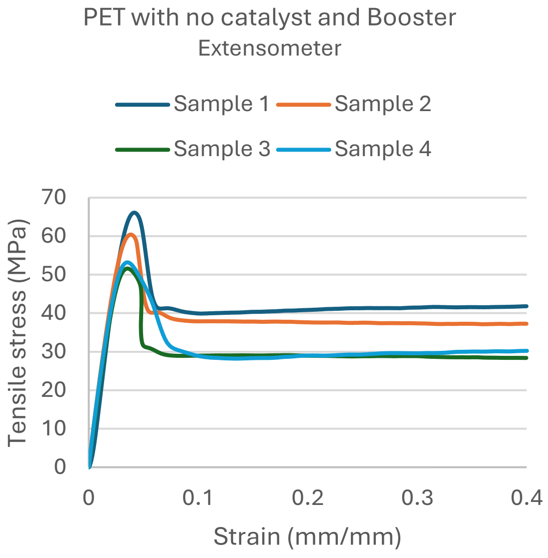

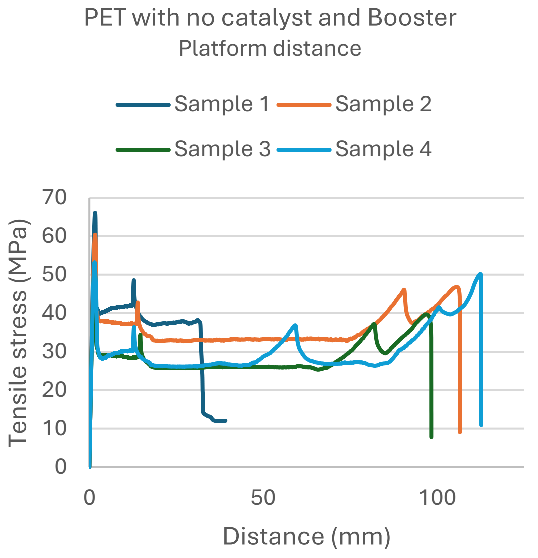

	Tensile Modulus (MPa)	Maximum Tensile stress (MPa)	Tensile stress at Yield Offset 0.002 mm/mm (MPa)	Extension at Break (Standard) (%)
Sample 1	2274	66.1	54.5	195.5%
Sample 2	2269	60.4	43.0	532.6%
Sample 3	2129	51.6	38.7	491.7%
Sample 4	2207	53.2	37.2	563.4%
Maximum	2274	66.1	54.5	563.4%
Mean	2220	57.8	43.4	445.8%
Minimum	2129	51.6	37.2	195.5%
Stdev.	59	6.7	7.8	169.5%

Raw data tensile testing of <u>commercial RAMA PET N180:</u>

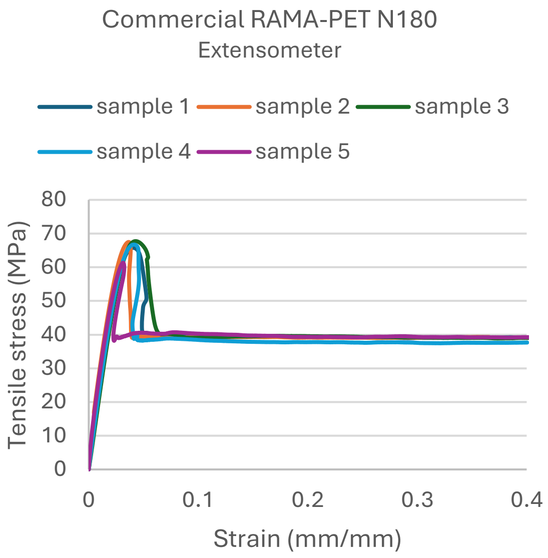

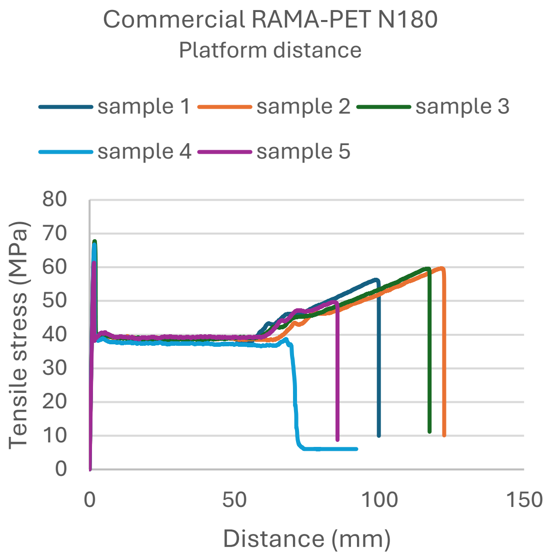

	Tensile Modulus	Maximum Tensile stress	Tensile stress at Yield	Extension at Break (Standard)
	(MPa)	(MPa)	Offset 0.002 mm/mm (MPa)	(%)
Sample 1	2477	65.8	43.3	499.2%
Sample 2	3222	67.5	35.9	612.0%
Sample 3	2296	67.7	54.8	586.8%
Sample 4	2409	66.7	46.9	370.4%
Sample 5	2705	61.3	33.9	427.9%
Maximum	3222	67.7	54.8	612.0%
Mean	2622	65.8	43.0	499.2%
Minimum	2296	61.3	33.9	370.4%
Stdev.	328	2.3	7.6	91.7%

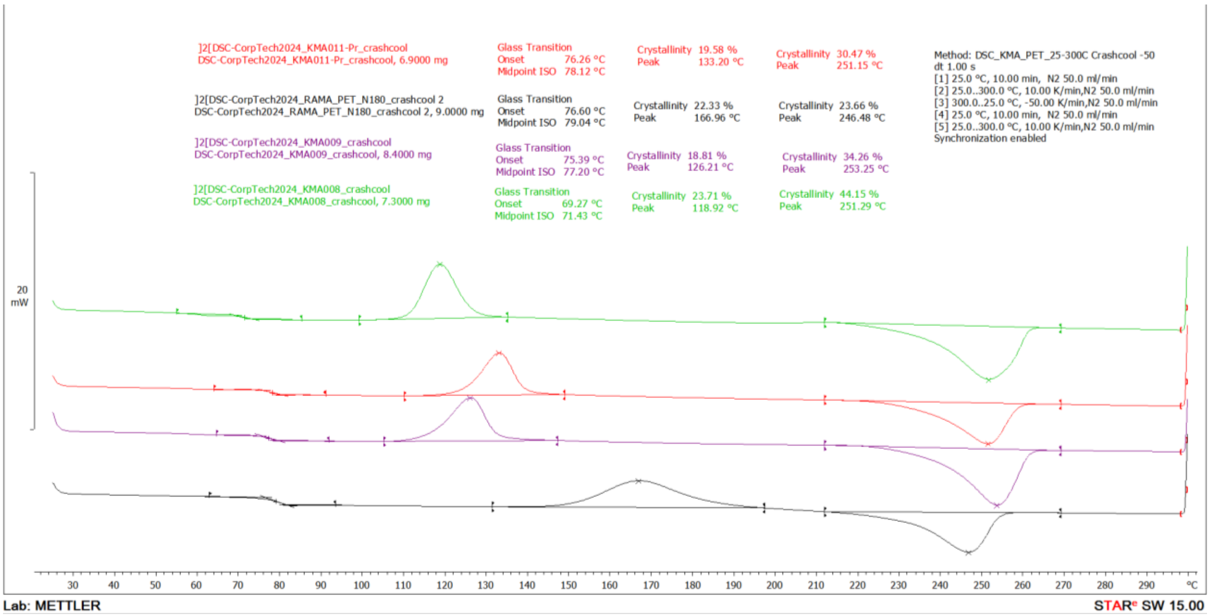

DSC of the PET produced without catalyst and the commercial RAMA PET

Top (red); *with* DGO, second (black); RAMA-PET, third (purple); *without* DGO, Fourth (green); esterification product prior to polycondensation. Commercial Rama PET also contains additives and a portion of isophthalic acid (4%), which should be kept in mind when comparing them to the synthesized polymers.

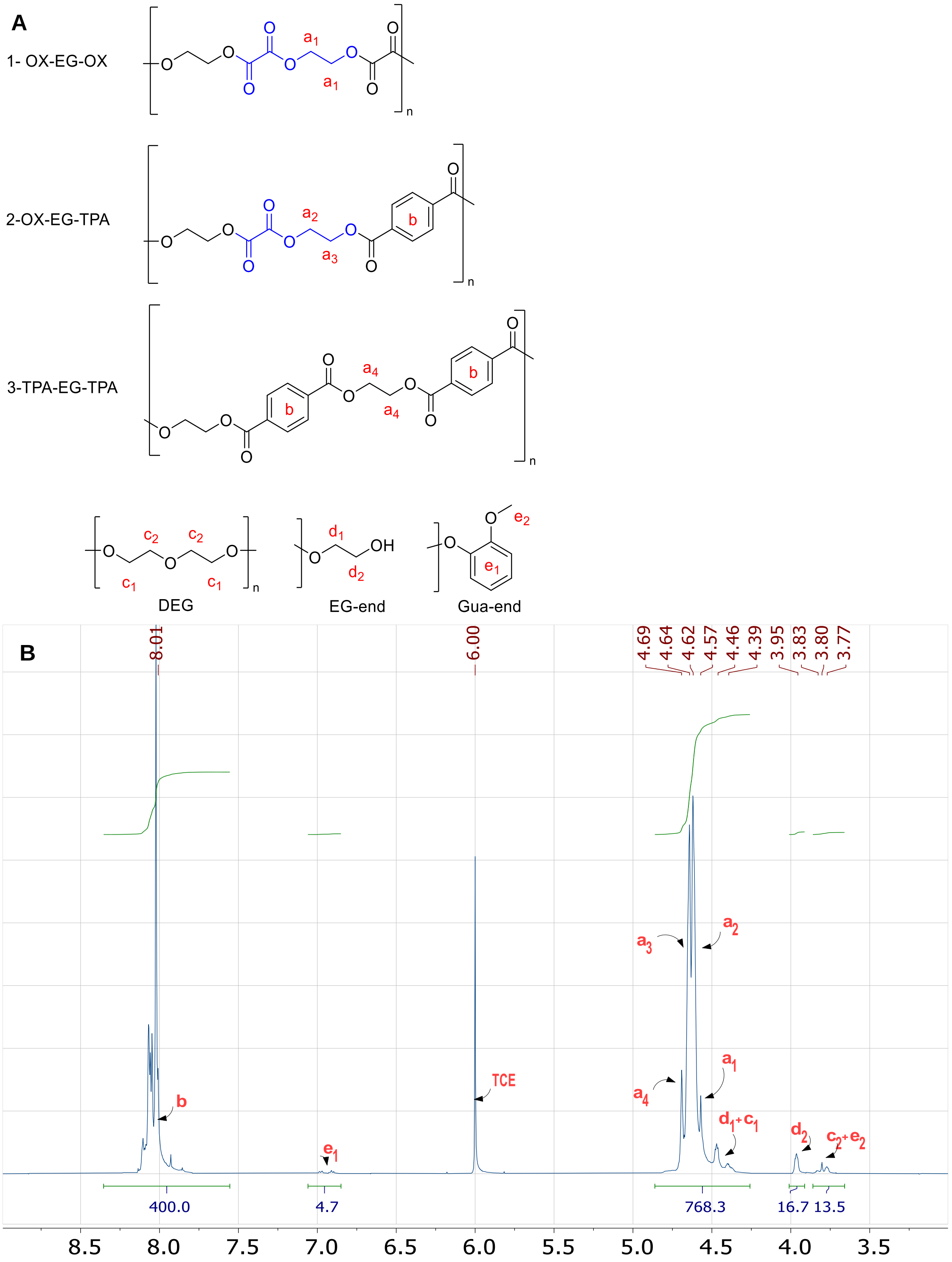

Fig.S1 | ^{1}H NMR of PETO with 48% oxalate in TCE-d_2

A, as PETO contains two diacids (TPA and oxalate), the sequence of the polymer backbone can be organized in various ways: at the top you can see the different polymer repeating structures of PETO with the presence of ethylene oxalate repeating unit highlighted.

B, The ^{1}H NMR of PETO with 48% oxalate in TCE-d_2; all of the signals could be assigned to the above mentioned PETO polymer structures. Since the diols reacted to create an ester with either terephthalate or oxalate (a_1, a_2, a_3, a_4, c_1, d_1), the amount of oxalate in the polymer can be quantified using the signals of terephthalate and the diols. See experimental section [B] for the synthesis details.

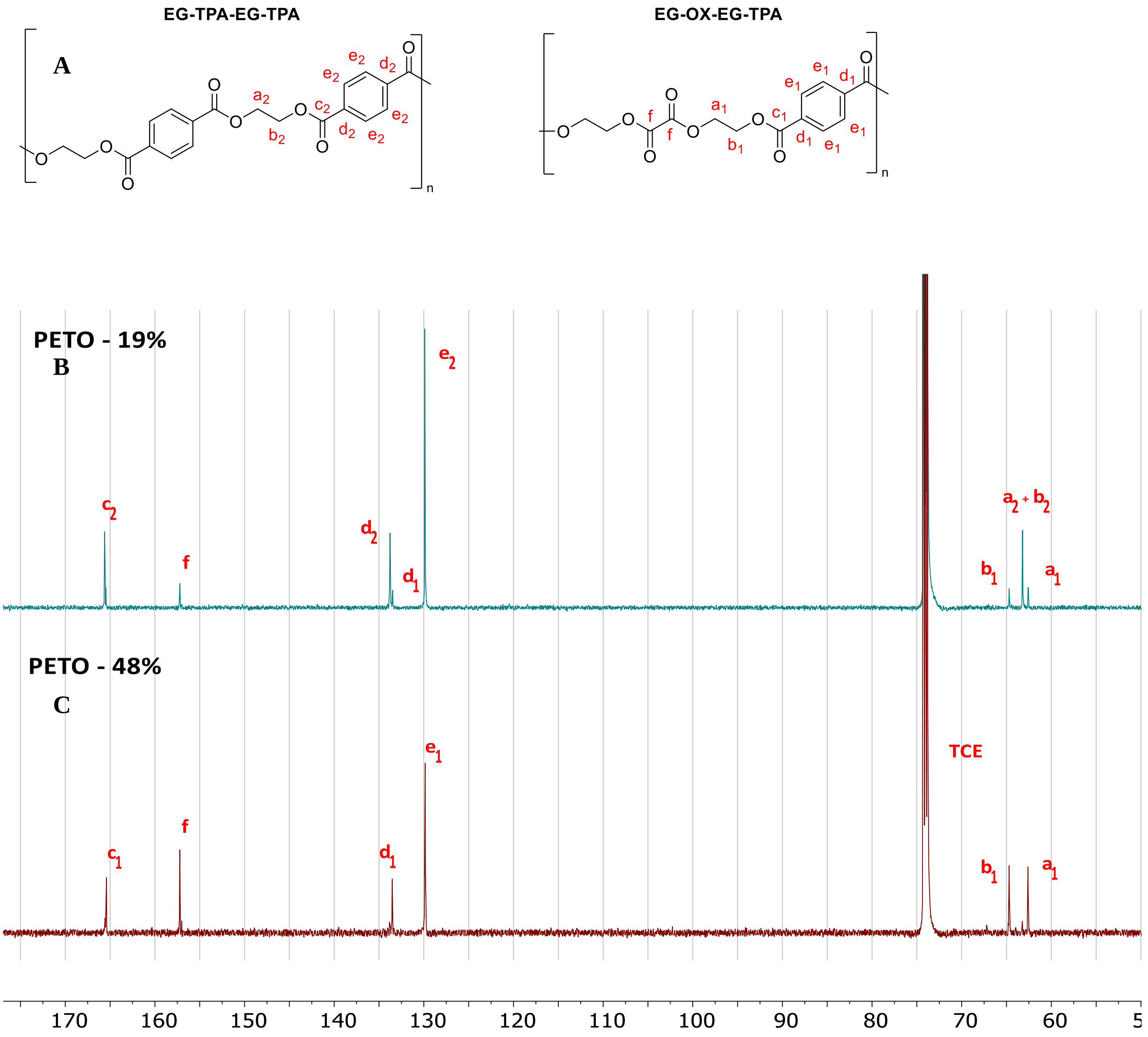

Fig.S2 | ^{13}C-NMR of PETO in TCE-d$_2$

A, the different polymer repeating structures of PETO with the carbons numbered for assigning the NMR. **B,** ^{13}C NMR of PETO with 19% oxalate. **C,** ^{13}C NMR of PETO with 48% oxalate. As seen with the ^{1}H NMR of PETO with 48% oxalate, the polymer backbone mainly consist of the alternating sequence OX-EG-TPA. Only small amounts of the other sequences are seen. Note that the oxalate signal shifts at 157.2 ppm. See experimental section [C] for the synthesis details.

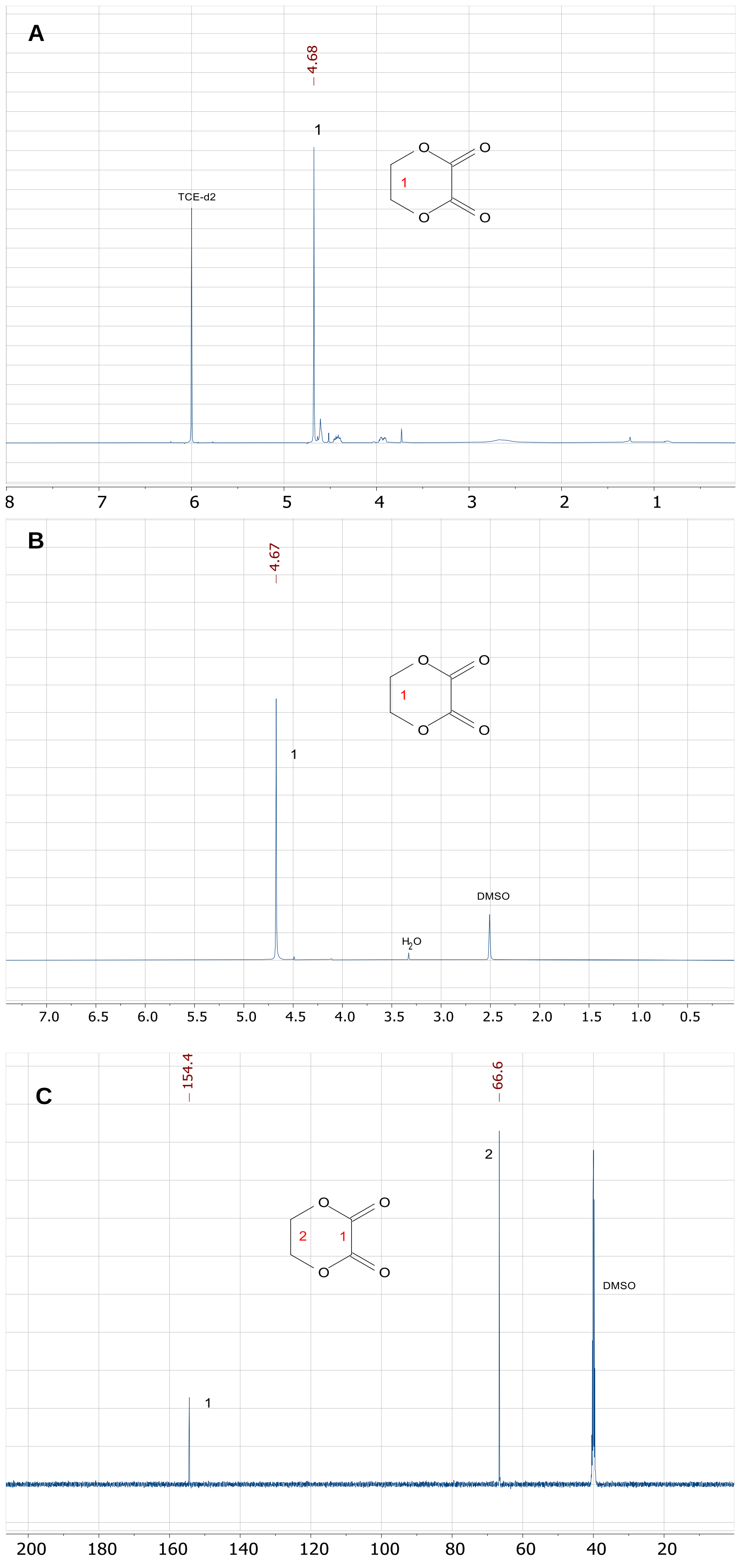
A
4.68
1
TCE-d2
O
O
O
O
1
8
7
6
5
4
3
2
1
B
4.67
1
O
O
O
O
1
DMSO
H2O
7.0
6.5
6.0
5.5
5.0
4.5
4.0
3.5
3.0
2.5
2.0
1.5
1.0
0.5
C
154.4
66.6
2
1
O
O
O
O
2
1
DMSO
200
180
160
140
120
100
80
60
40
20

Fig.S3 | NMR spectra of ethylene oxalate ring (1,4-Dioxane-2,3-dione). A, ^{1}H NMR of EO in TCE-d_2, note that Ethylene oxalate was only soluble in TCE-d_2 upon strong heating, causing some new signals to form, likely polyethylene oxalate formed. **B,** ^{1}H NMR of EO in DMSO-d_6 **C,** ^{13}C NMR of EO in DMSO-d_6.

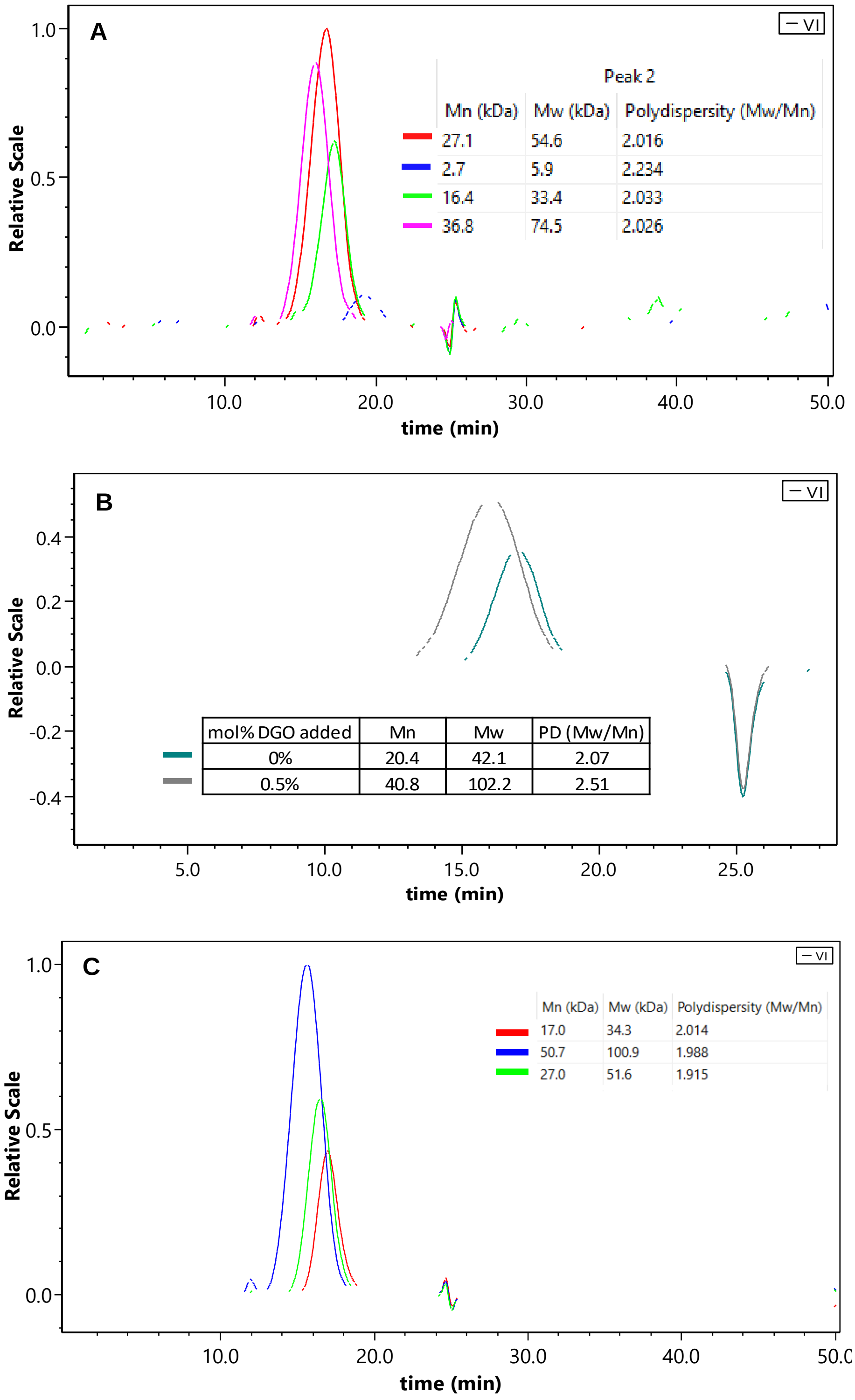

Fig.S4 | SEC results small scale polymerizations
A, SEC results of PET melt polycondensate without catalyst. Blue; esterification product, green; without DGO, Red; with DGO, pink; commercial SSP RAMAPET N180
B, SEC- DGO to a melt polycondensate of PET (with titanium catalyst)
C, SEC results DGO portion wise to a melt polycondensate of PEF and SSP (without catalyst)

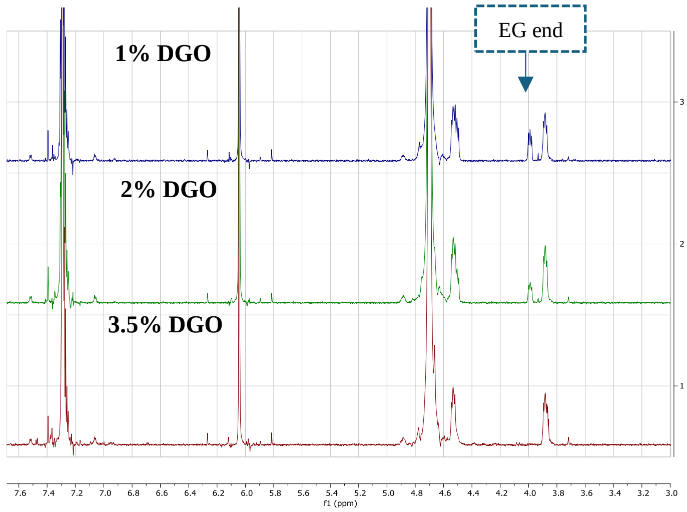

DGO (mol%)	EG end (mol%)	M_n (kDa)	M_w (kDa)	PDI (M_w/M_n)
1	3.5	17.0	34.3	2.0
2	2.3	27.0	51.6	1.9
3.5	<0.1	50.7	100.9	2.0

Fig.S5 | Stepwise addition of DGO to a post polycondensation of PEF. After each addition (1, 2, 3.5 mol%) ^{1}H NMR and SEC of the resulting polymer were taken. The resulting SEC molecular weights and ethylene glycol end groups are shown in the table. See experimental section [H] for the synthesis details.

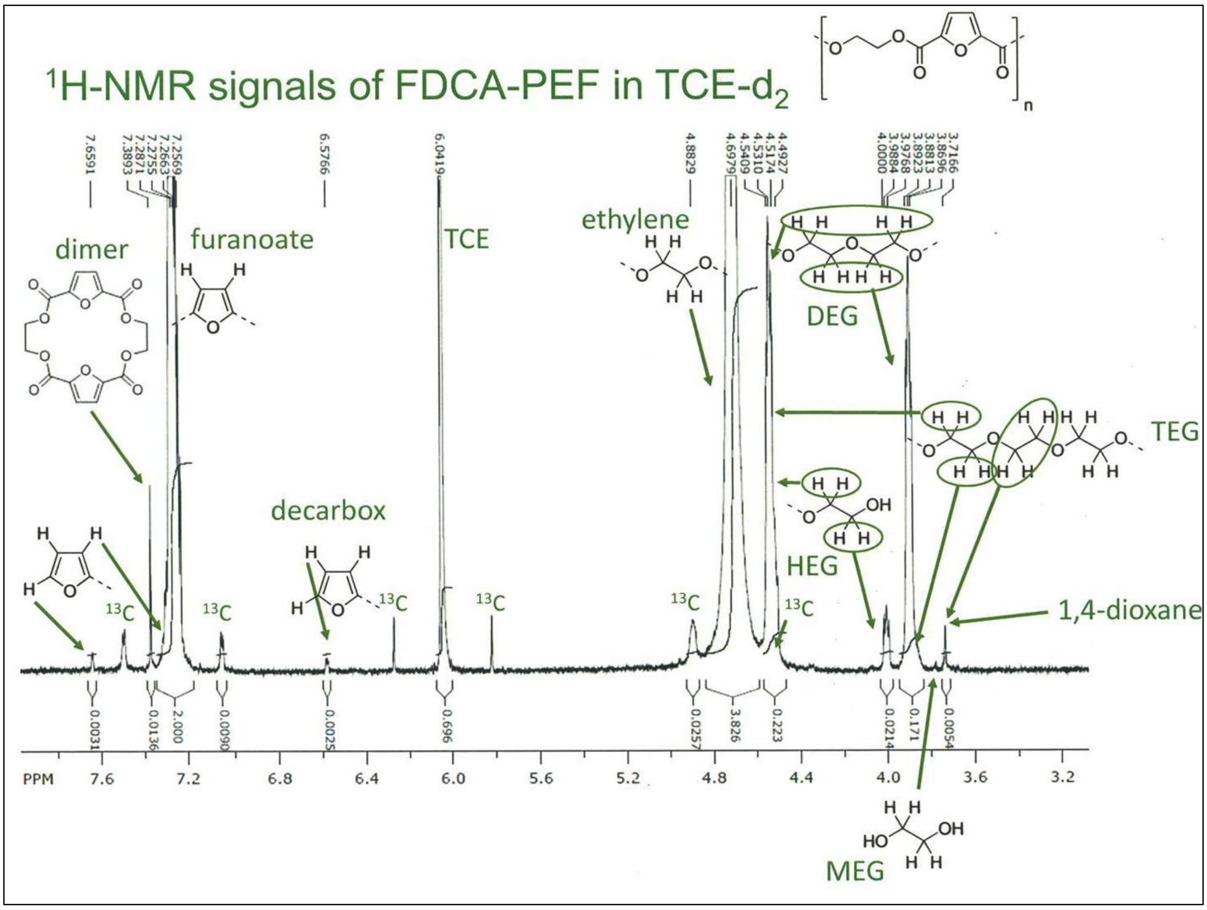

Fig.S6 | ^{1}H-NMR of PEF (produced via FDCA) in TCE-d$_2$. The corresponding signals of PEF and common side products are assigned. Note that the decarboxylation of FDCA can be observed by the shift at 6.58 ppm when the TCE residual solvent shift is set at 6.04 ppm. According to the NMR integrals, the DEG content of the polymer is 4.1% (based on total FDCA). See experimental section [G] for the synthesis details.

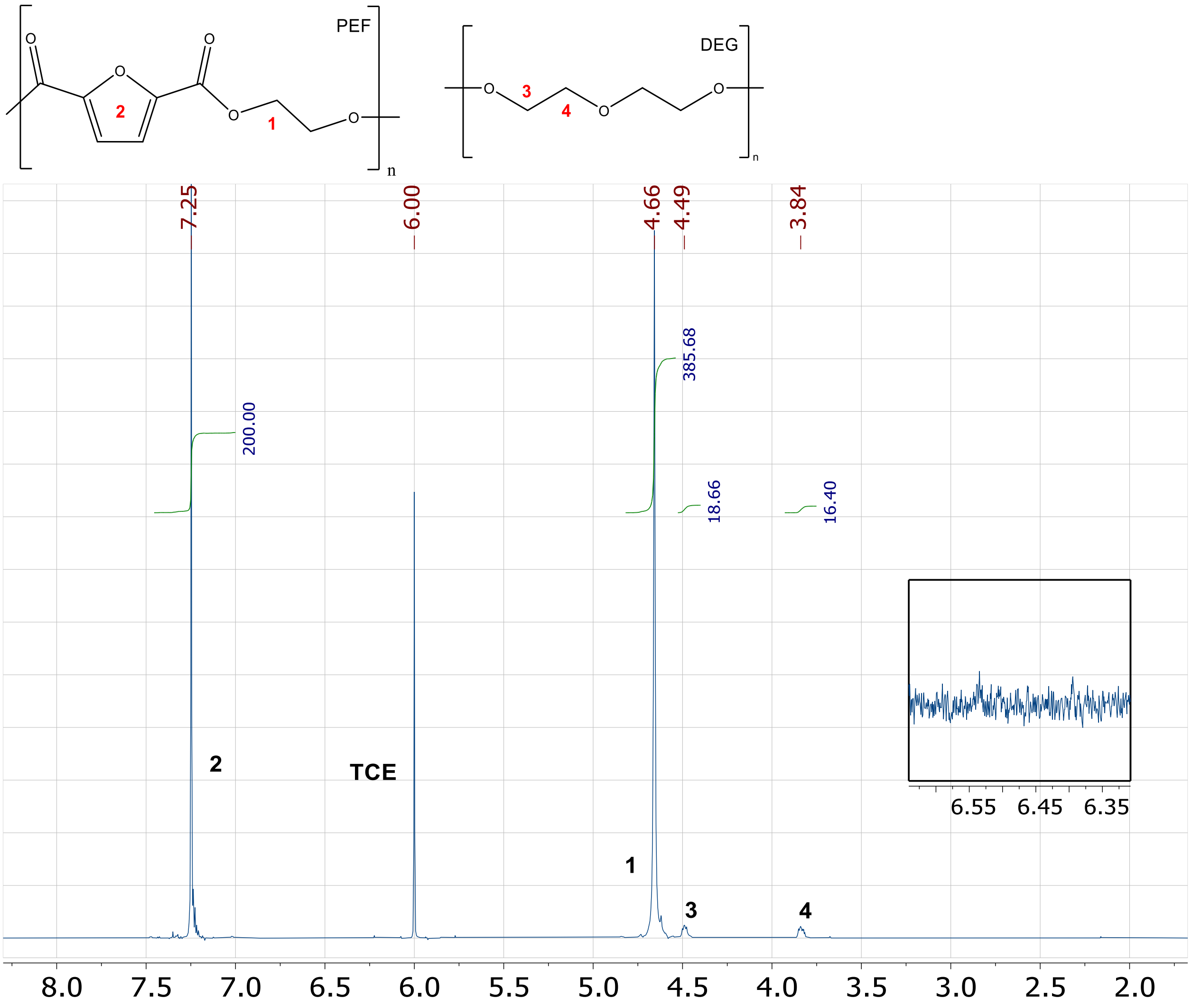

Fig.S7a | ^{1}H-NMR of PEF after stepwise addition of 3.5 mol% DGO.
The FDCA decarboxylation is highlighted; 6.54 ppm in TCE-d_2 (residual solvent signal is set at 6.00 ppm). The decarboxylation is likely reduced by the absence of a catalyst and mild conditions. According to the NMR integrals, the DEG content of the polymer is 4.1% (based on total FDCA). See experimental section [H] for the synthesis details.

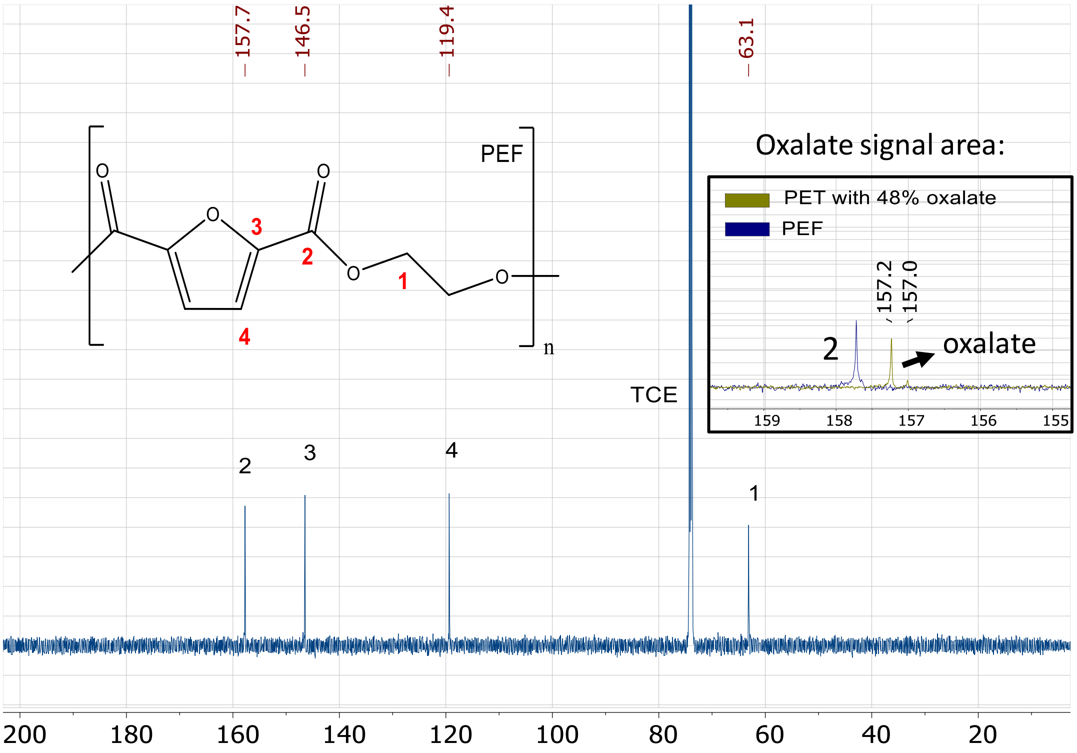

Fig.S7b | ^{13}C NMR of PEF after stepwise addition of 3.5 mol% DGO.
The expected region where the oxalate would show up highlighted by overlaying the ^{13}C NMR of PETO (157.0 ppm). No oxalate is observed. See experimental section [H] for the synthesis details.

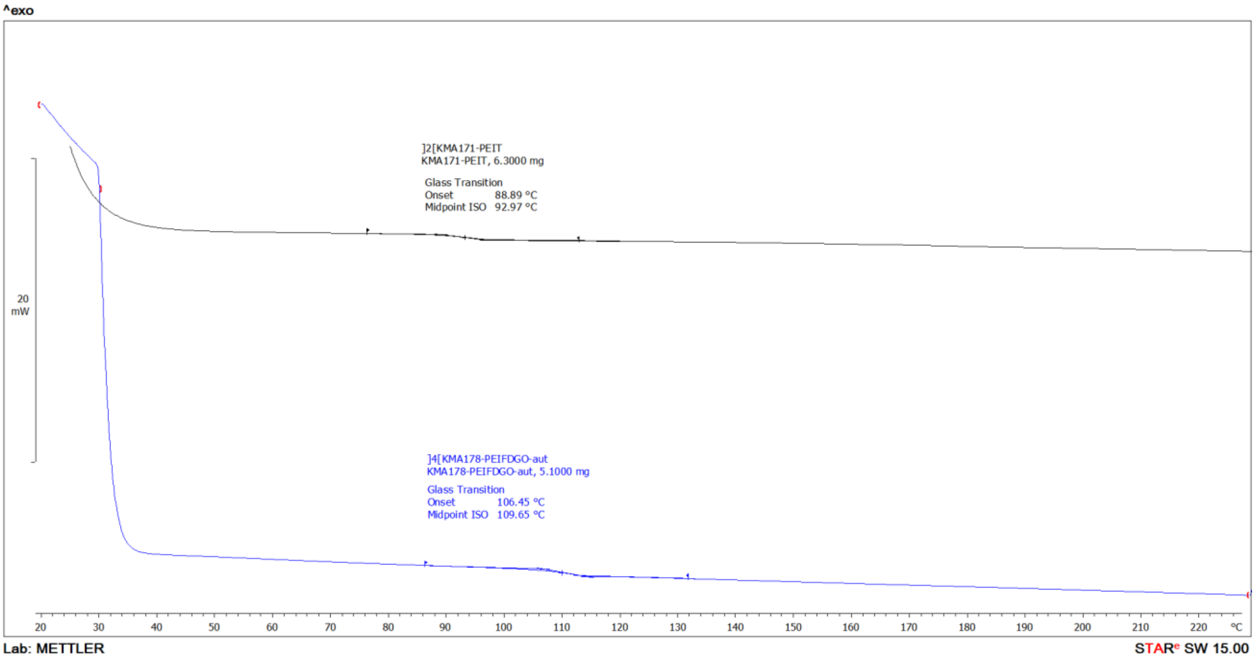

Fig.S8 | DSC of PEIF and PEIT produced in the autoclave.
The black line represents the PEIT with 16% isosorbide and has a T_g of 93 °C. The blue line represents the PEIF with 26.3% isosorbide and has a T_g of 110 °C.

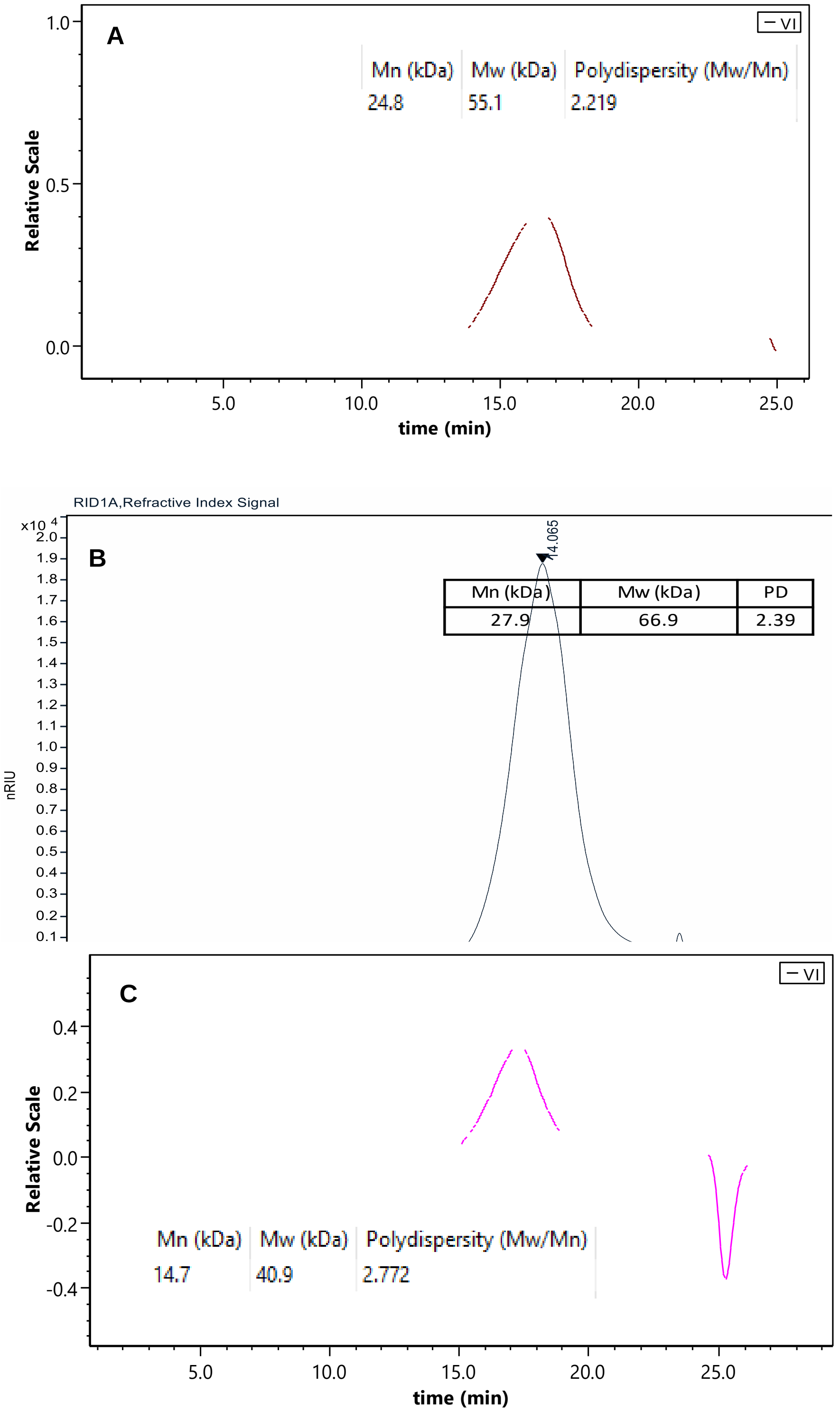

Fig.S9 | SEC of PEIT and PEIF
A, SEC of the PEIT made in the 2L autoclave, final product.
B, SEC of the PEIF made in the 2L autoclave, final product.
C, SEC of the PEIT filament made with the 3Devo extruder.

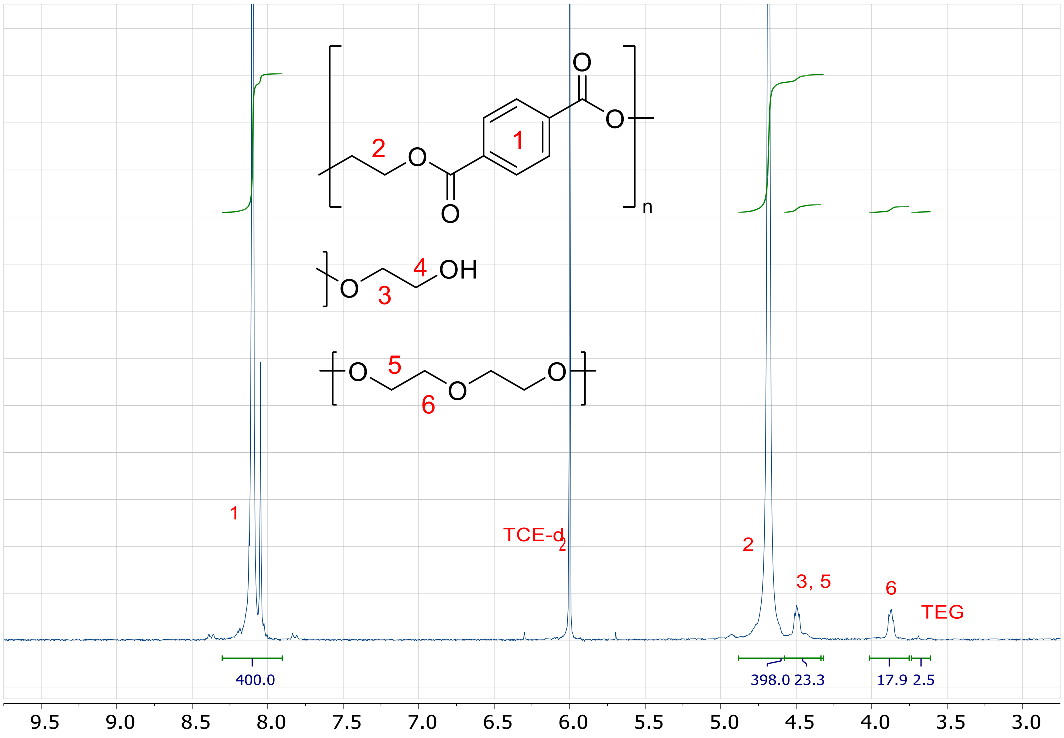

Fig.S10 | ^{1}H-NMR of PET without booster in TCE-d₂

According to the NMR integrals, the DEG content of the polymer is 4.5 mol% relative to the total TPA content. A part (0.7 mol%) of this DEG content comes from the starting material BHET, which contains 1.3 mol% DEG (see supplementary information). See experimental section [D] and [E] for the synthesis details.

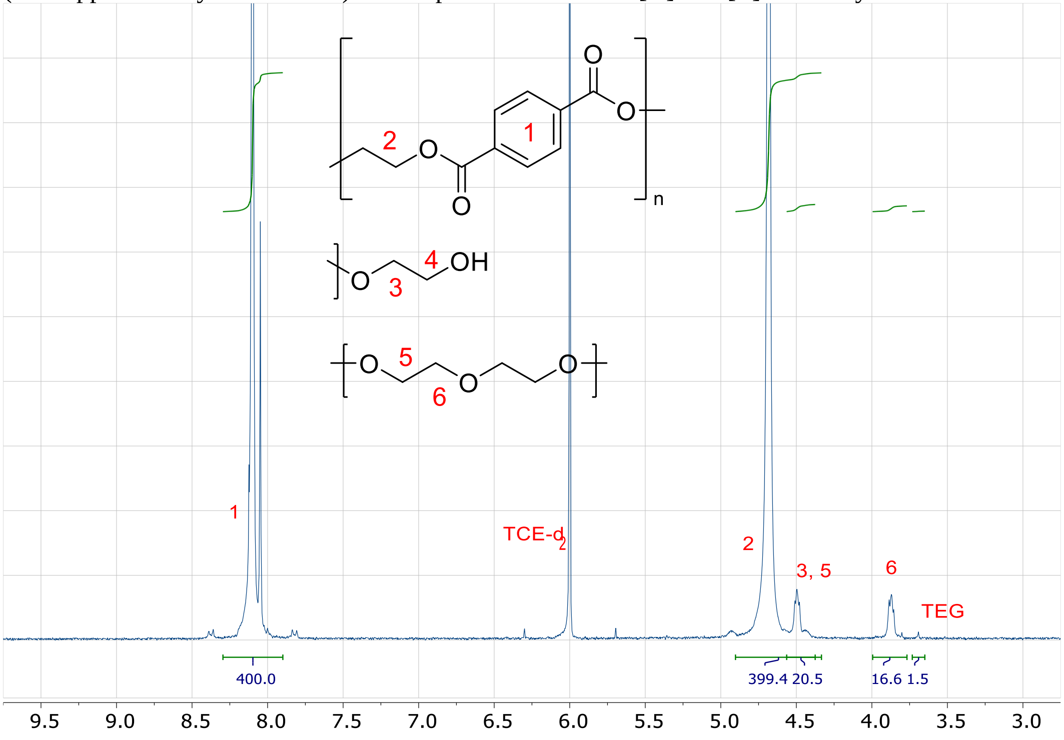

Fig.S11 | ^{1}H-NMR of PET with 2.2 mol% booster in TCE-d₂.

According to the NMR integrals, the DEG content of the polymer is 4.2 mol% relative to the total TPA content. A part (0.7 mol%) of this DEG content comes from the starting material BHET, which contains 1.3 mol% DEG (see supplementary information **Overview S4**). See experimental section [D] and [E] for the synthesis details**.**

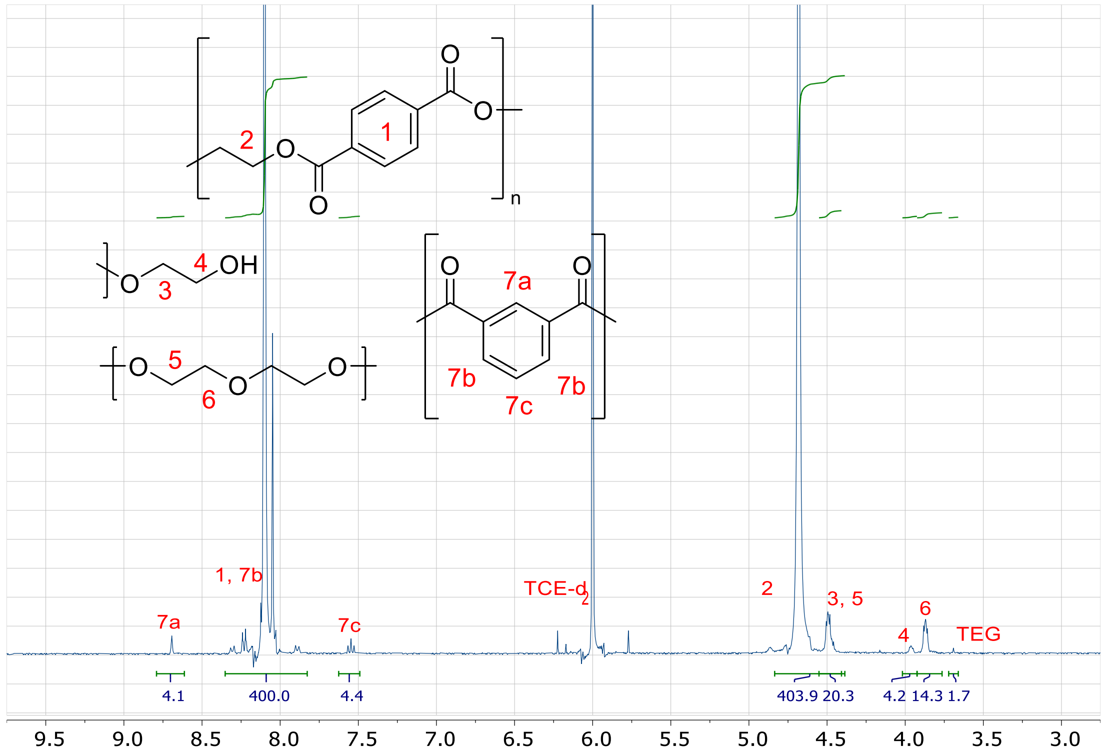

Fig.S12 | ^{1}H-NMR of commercial RAMAPET N180 in TCE-d$_2$ (IV: 0.8 dl/g)

According to the NMR integrals, the DEG content of the polymer is 3.6 mol% relative to the total TPA content. Signals 7a and 7b show that the commercial Rama PET also contains a portion of isophthalic acid (~4%), which should be kept in mind when comparing them to the synthesized polymers.

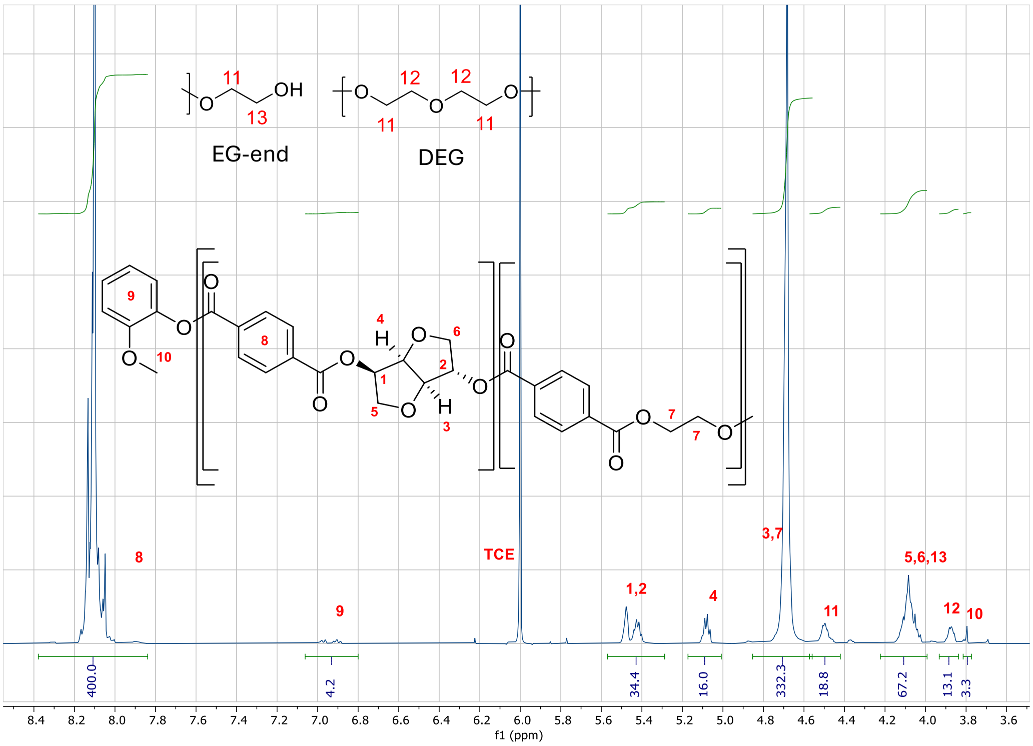

Fig.S13 | ^{1}H-NMR of PEIT in TCE-d₂ produced by the autoclave experiment.
According to the NMR integrals, the DEG content of the polymer is 3.3% and the isosorbide content is 16%, both are percentage of the total TPA content. A part of this DEG content comes from the starting material BHET, which contains 1.3 mol% DEG (see supplementary information **Overview S4**). See experimental section [I] for the synthesis details. Ratio TPA : diol = $\frac{\mathbf{332.3-16.0}}{\mathbf{4}} + \frac{\mathbf{16.0}}{\mathbf{1}} + \frac{\mathbf{18.8}}{\mathbf{4}}$ = 1.00 : 0.998. 6 mol% of DGO was added, if all oxalate would be incorporated into the polymer the TPA : diol ratio would be 1.00 : 1.064.

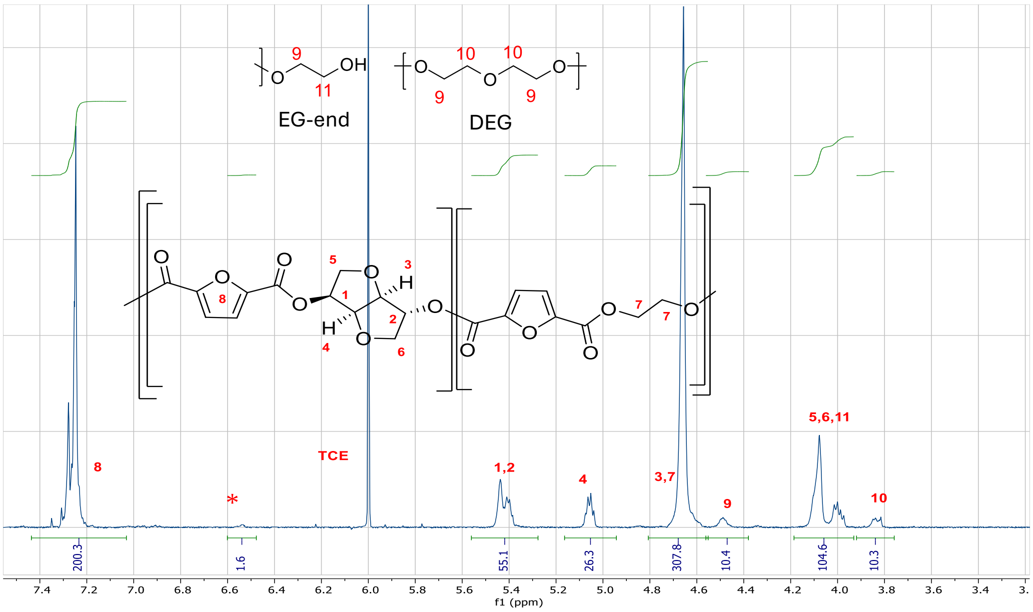

Fig.S14 | ^{1}H-NMR of PEIF in TCE-d₂ produced by the autoclave experiment.
* indicates the decarboxylation of FDCA. According to the NMR integrals, the DEG content of the polymer is 2.6% and the isosorbide content is 26.3%, both are percentage of the total FDCA content. See experimental section [J] for the synthesis details. Ratio FDCA : diol = $\frac{\mathbf{307.8-26.3}}{\mathbf{4}} + \frac{\mathbf{26.3}}{\mathbf{1}} + \frac{\mathbf{10.4}}{\mathbf{4}}$ = 1.00 : 0.993. 5.2 mol% of DGO was added, if all oxalate would be incorporated into the polymer the FDCA : diol ratio would be 1.00 : 1.055.

Overview S4 | additional experimental results and analysis

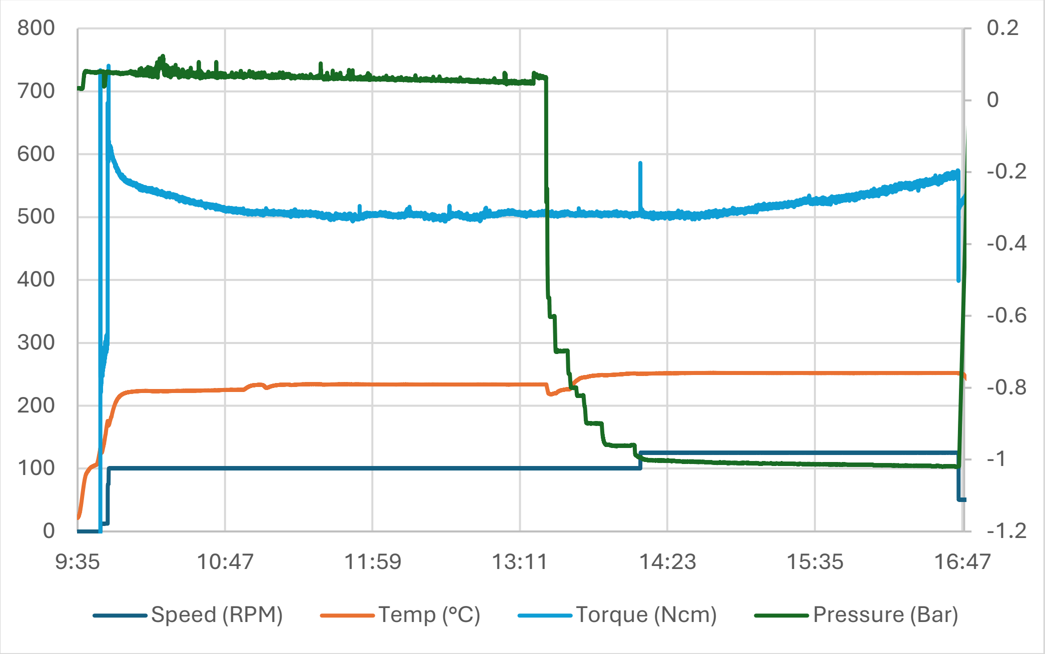

The autoclave parameter readout for the PEIT polymerization on day 1, experimental section [I]. The temperature, speed, and torque are plotted on the left Y-axis, while pressure is scaled on the right Y-axis.

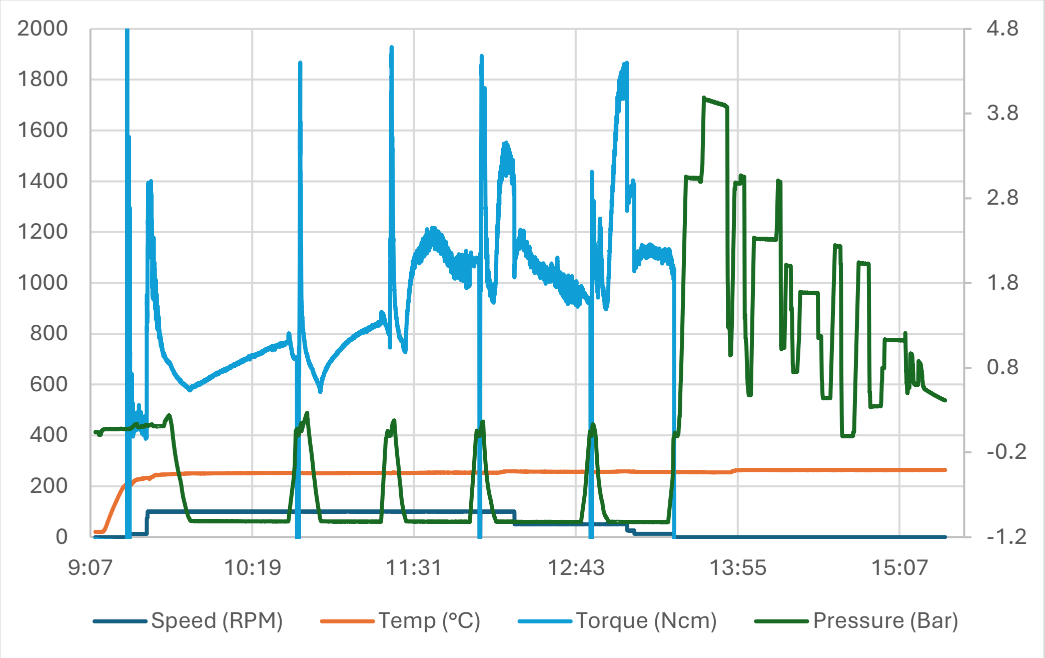

The autoclave parameter readout for the PEIT polymerization on day 2, experimental section [I]. The temperature, speed, and torque are plotted on the left Y-axis, while pressure is scaled on the right Y-axis.

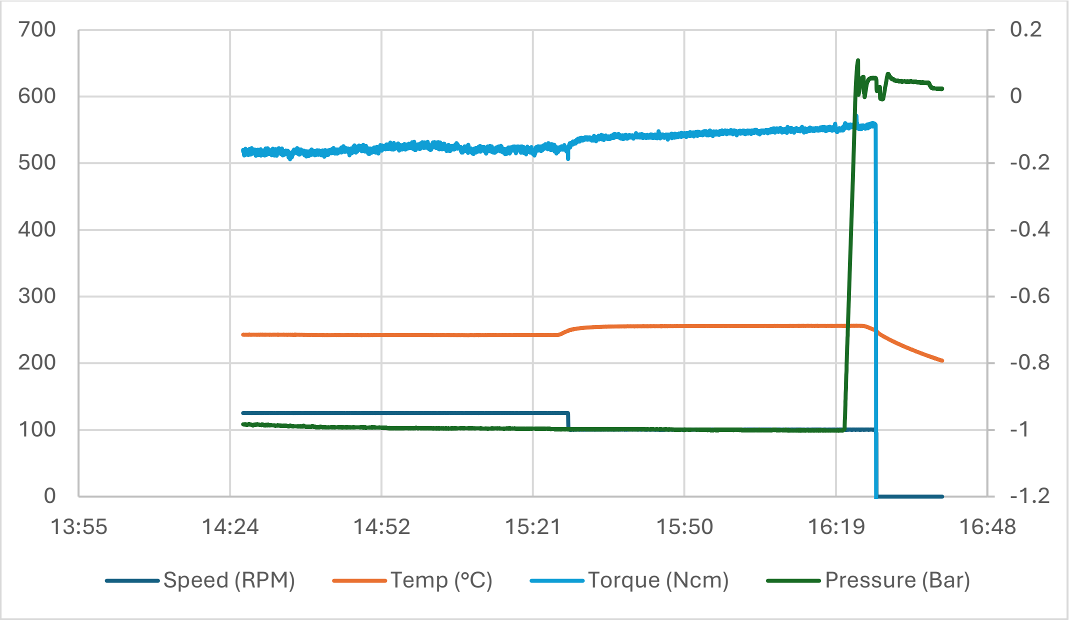

The autoclave parameter readout for the PEIF polymerization on day 1, experimental section [F]. The temperature, speed, and torque are plotted on the left Y-axis, while pressure is scaled on the right Y-axis. Day 1 was only partially tracked due to a computer malfunction.

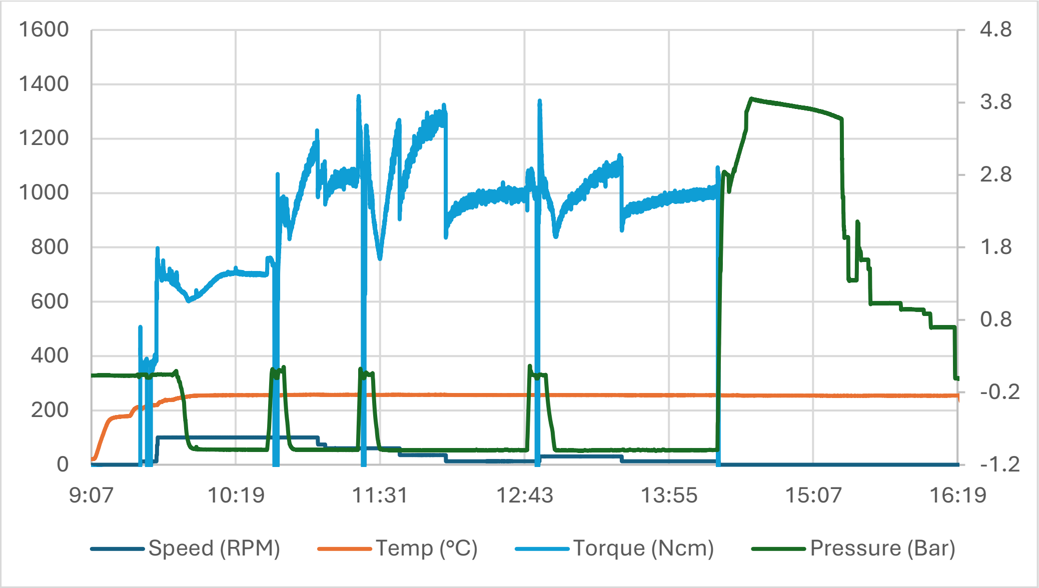

The autoclave parameter readout for the PEIF polymerization on day 2, experimental section [F]. The temperature, speed, and torque are plotted on the left Y-axis, while pressure is scaled on the right Y-axis.

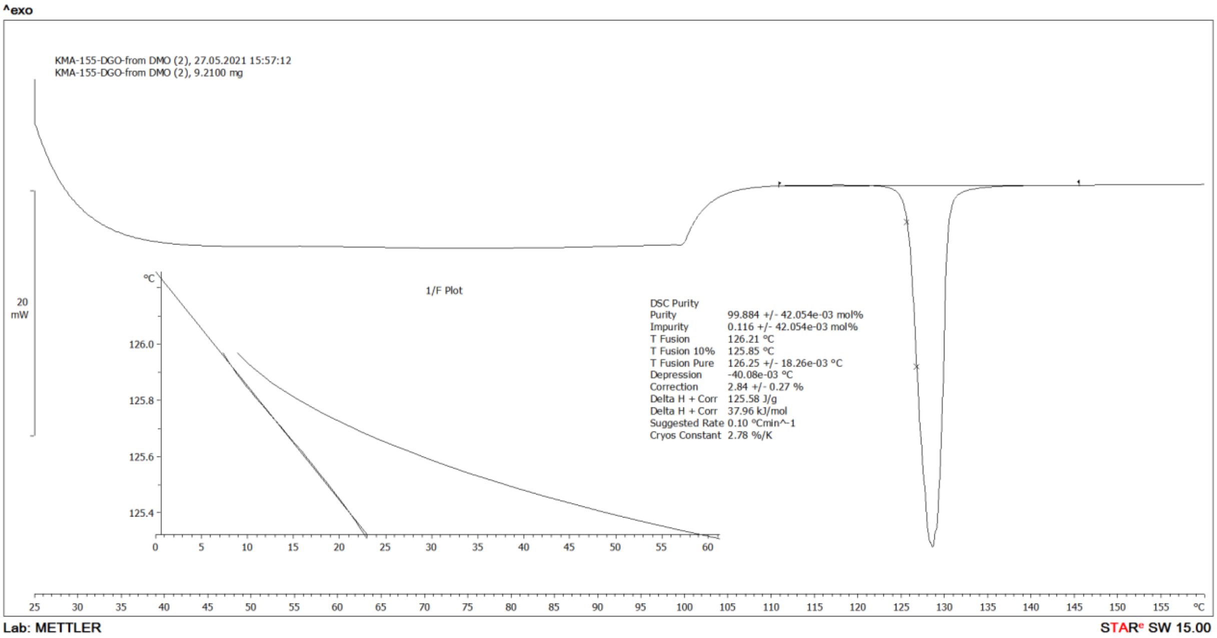

DSC melting curve of DGO from Dimethyl oxalate with purity analysis. A melting point of 126.2°C is observed. As the correction factor for the purity analysis is lower than 5% we can accept the value of 99.9 mol%. However, this value excludes the impurities which are insoluble in the melt. See experimental section [A] for the synthesis details.

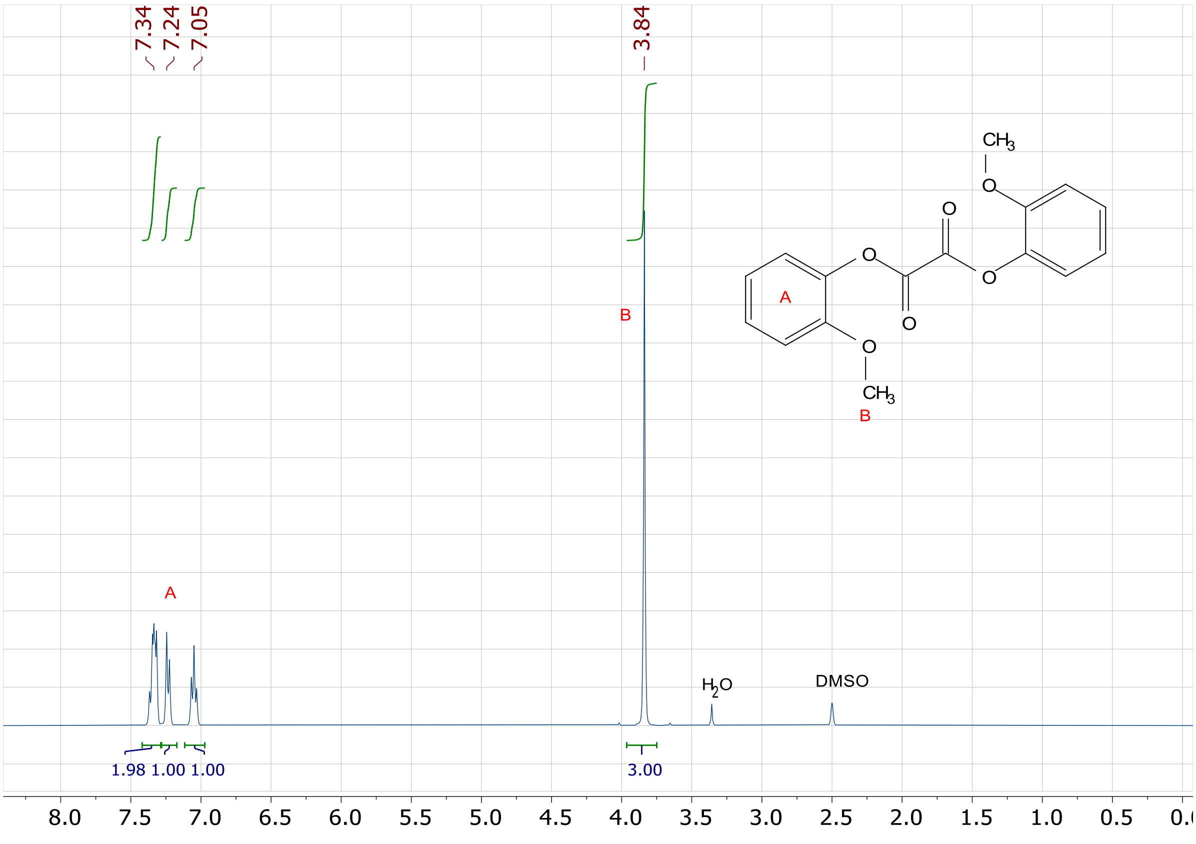

^{1}H NMR of DGO in DMSO-d$_6$. The signals and integrals align with the structure of DGO and are consistent with the spectra reported in the literature.[59] See experimental section [A] for the synthesis details.

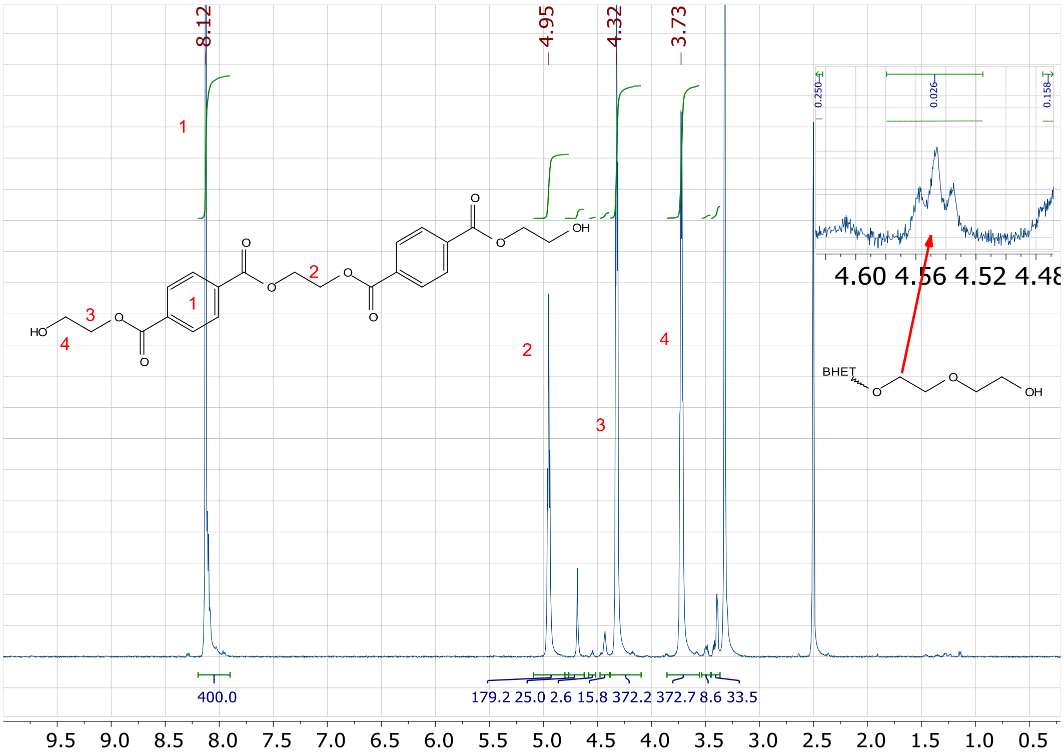

^{1}H NMR of BHET in DMSO-d$_6$. The BHET contains 1.3 mol% DEG based on total TPA, see integral at 4.55 ppm (DMSO-d$_6$).[60]

references

1 Z. S. Şahin, G. K. Kantar, S. Şaşmaz and O. Büyükgüngör, Synthesis, molecular structure, spectroscopic analysis, thermodynamic parameters and molecular modeling studies of (2-methoxyphenyl) oxalate, *J. Mol. Struct.*, 2015, **1087**, 104–112, DOI: 10.1016/j.molstruc.2015.01.039.

2 W. K. Son, K. H. Lee, K. S. Kang, Y. H. Kim and H. M. Jung, Siontech Co Ltd [KR], Method for chemical recycling of glycol modified polyethylene terephthalate wastes, 2018, 101888612BB1
